# Integration of modeling with experimental and clinical findings synthesizes and refines the central role of inositol 1,4,5-trisphosphate receptor 1 in spinocerebellar ataxia

**DOI:** 10.3389/fnins.2014.00453

**Published:** 2015-01-21

**Authors:** Sherry-Ann Brown, Leslie M. Loew

**Affiliations:** ^1^Department of Medicine, Mayo ClinicRochester, MN, USA; ^2^Richard D. Berlin Center for Cell Analysis and Modeling, University of Connecticut Health CenterFarmington, CT, USA

**Keywords:** spinocerebellar ataxia, translational, model, computational, inositol 1,4,5-trisphosphate receptor 1, Purkinje, carbonic anhydrase related proteins, homer

## Abstract

A suite of models was developed to study the role of inositol 1,4,5-trisphosphate receptor 1 (IP3R1) in spinocerebellar ataxias (SCAs). Several SCAs are linked to reduced abundance of IP3R1 or to supranormal sensitivity of the receptor to activation by its ligand inositol 1,4,5-trisphosphate (IP3). Detailed multidimensional models have been created to simulate biochemical calcium signaling and membrane electrophysiology in cerebellar Purkinje neurons. In these models, IP3R1-mediated calcium release is allowed to interact with ion channel response on the cell membrane. Experimental findings in mice and clinical observations in humans provide data input for the models. The SCA modeling suite helps interpret experimental results and provides suggestions to guide experiments. The models predict IP3R1 supersensitivity in SCA1 and compensatory mechanisms in SCA1, SCA2, and SCA3. Simulations explain the impact of calcium buffer proteins. Results show that IP3R1-mediated calcium release activates voltage-gated calcium-activated potassium channels in the plasma membrane. The SCA modeling suite unifies observations from experiments in a number of SCAs. The cadre of simulations demonstrates the central role of IP3R1.

## Introduction

**Figure 1:**
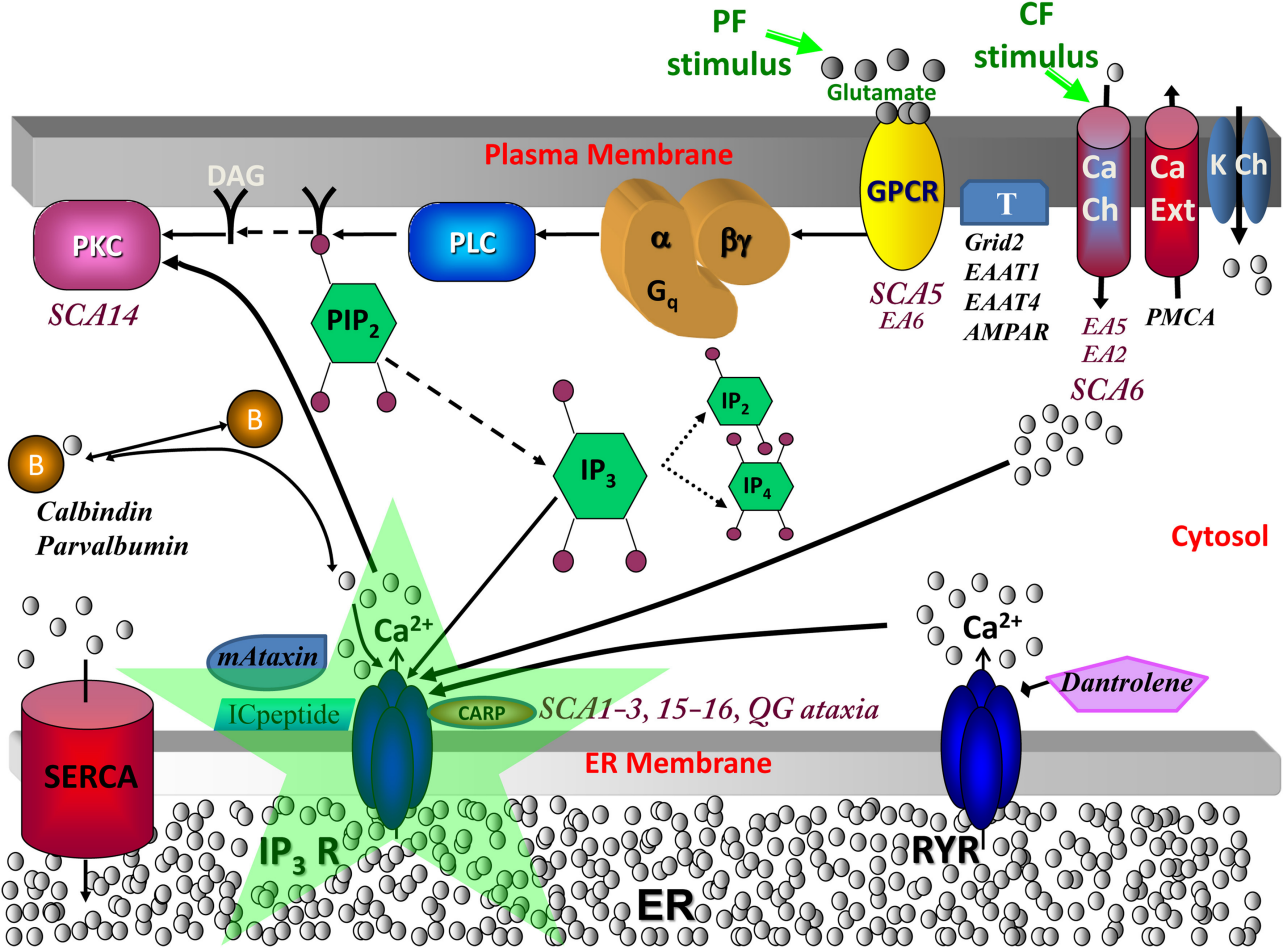
**Calcium signaling in cerebellar Purkinje neuron spiny dendrites with various molecules involved in spinocerebellar ataxias**. When a parallel fiber (PF) stimulates a distal dendrite of the Purkinje neuron, the neurotransmitter glutamate is released and binds its receptor, metabotropic glutamate receptor (mGluR; from the group C family of G protein-coupled receptors, GPCR), on the plasma membrane of the Purkinje dendrite (Finch and Augustine, 1998; Takechi et al., 1998). This leads to the activation of phospholipase C (PLC), a hydrolyzing enzyme that cleaves PIP2 in the plasma membrane to produce diacylglycerol (DAG) and IP3 (Cohen, 2003). DAG stays in the membrane, where it can activate cytosolic molecules close to the membrane, such as PKC (Nishizuka, 1984). IP3 diffuses away from the reaction site (the plasma membrane) to its receptor on the endoplasmic reticulum (ER) (Berridge et al., 2000). IP3 is also free to diffuse out of the spine head, through the spine neck, into the adjacent dendrite, and along the length of dendrite nearby, part of the dendritic reservoir. At the ER, IP3 binds its receptor, IP3R; the channel opens, releasing calcium from the internal store into the cytosol. This rise in calcium can activate calcium-dependent proteins, including calmodulin, various kinases (including PKC), and other enzymes. The climbing fiber (CF) stimulus depolarizes the entire neuron membrane, opening the voltage-gated calcium channels (Ito et al., 1982). The calcium that flows into the Purkinje neuron spine can also bind IP3R1, along with IP3 from parallel fiber activation of the spine, leading to supralinear calcium release. SCA, spinocerebellar ataxia; EA, episodic ataxia; QG, quadrupedal gait ataxia; K Ch, potassium channels including Kv1.1 (mutated in Episodic Ataxia type 1; Imbrici et al., 2003), Kv3.3 (mutated in SCA13; Waters et al., 2006), Kv4.3 (mutated in SCA1; Duarri et al., 2012 and SCA22 Lee et al., 2012), BK and SK, which are respectively, the large (big) conductance calcium-activated voltage-gated potassium channel which is the potassium channel involved in Purkinje membrane repolarization, and the small conductance calcium-activated voltage-independent potassium channel; DAG, diacylglycerol, which is a product of PLC hydrolysis that along with calcium co-activates PKC; PIP2, Phosphatidylinositol 4,5-bisphosphate, a plasma membrane phospholipid of the inner leaflet that gives rise to DAG and IP3 when hydrolyzed; PLC, phospholipase C, an enzyme that hydrolyzes PIP2 when activated by G-βγ from mGluR; mGluR or Grm1, metabotropic glutamate receptor type 1 (Guergueltcheva et al., 2012); T, other glutamate transporters and receptors including Grid2, Excitatory amino-acid transporter type 1 (EAAT1; mutated in Episodic Ataxia type 6, de Vries et al., 2009), Excitatory amino-acid transporter type 4 (EAAT4; Spectrin β, an anchor for EAAT4 and Grm1 is mutated in SCA5, Ikeda et al., 2006), and AMPAR; B, calcium-binding proteins or buffers including calbindin and parvalbumin (Supplemental Material, S1 Calcium buffers in SCA and Supplemental Figure S2); mAtaxin, mutant ataxin proteins including Ataxin-1 through Ataxin-7; ICpeptide, peptides that resemble the tip of IP3R1 and thereby competitively bind mAtaxin; SERCA, sarcoendoplasmic reticulum calcium ATPase, a transporter for calcium entry from the cytosol to the smooth endoplasmic reticulum (ER); CARP, Carbonic anhydrase-related protein (particularly CARP VIII), an IP3R1 antagonist (Türkmen et al., 2009) (Supplemental Material, S4 IP3R1 suppression by CARP); RYR, ryanodine receptor, a transporter of calcium from the ER to the cytosol in dendrites (but not present in spines) in response to binding of specific ligands such as ryanodine; Dantrolene, a drug that inhibits RYR (Supplemental Material, S2 Calcium-induced calcium release crosstalk); PMCA, Plasma membrane calcium ATP-ase transports calcium out of the cell; Ca Ch, calcium channels including store-operate channels (SOC) for store-operated calcium entry and Cav2.1, which is the main P-type calcium channel in PCs with nonsense/missense mutations causing episodic ataxia type 2, expansion of CAG repeats causing SCA6, and mutations in CavB4 an accessory subunit for Cav2.1 causing EA5 (Escayg et al., 2000); IP3R, inositol trisphosphate receptor (mutated in SCA15/16 and altered sensitivity in SCA1-3, antagonized in QG ataxia), intracellular calcium release channel on the endoplasmic reticulum gated by IP3; PKC, protein kinase C (mutated in SCA14) expressed in Purkinje neurons helps control expression of surface molecules including AMPAR. Adapted from Hernjak et al. (2005).

Several classes of spinocerebellar ataxia in humans and mice are associated with reduced IP3R1 levels (Matsumoto et al., 1996; Street et al., 1997; Zecevic et al., 1999; Lin et al., 2000; Ogura et al., 2001; Serra et al., 2004; Kurnellas et al., 2007; van de Leemput et al., 2007; Chou et al., 2008; Novak et al., 2010b; Castrioto et al., 2011; Marelli et al., 2011; Huang et al., 2012; Obayashi et al., 2012; Hansen et al., 2013; Sugawara et al., 2013) or increased sensitivity of the receptor to IP3 (Inoue et al., 2001; Chen et al., 2008; Liu et al., 2009), or both. Such disorders may be termed “IP3R1-associated ataxias” (Brown and Loew, 2009, 2012), which also encompasses ataxias with yet undetermined IP3R1 characteristics (e.g., Huang et al., 2012; Conroy et al., 2014). Beyond IP3R1-associated ataxias, a vast majority of ataxias converge on IP3R1-dependent signaling (Figure 1). Schorge et al. argue that the unifying feature of many cerebellar ataxias is their impact on IP3R1 (Schorge et al., 2010).

The SCA modeling suite is a collection of well-mixed (compartmental) and spatial (1D, 3D) computational models that simulate the biochemical and electrophysiological properties of the cerebellar Purkinje neuron involving various calcium signaling and ion channel molecules in constructed or experimentally derived geometries (Supplemental Material, Supplemental Figure S1 and Supplemental Table S1). The SCA suite examines the role of IP3R1 in SCA pathophysiology (Brown and Loew, 2012), with potential for translational studies.

## Novel predictions from the SCA modeling suite

### Coincidence detection time window at spine IP3R1

Cerebellar Purkinje neurons receive input from more than 150,000 granule cell axons (parallel fibers), leading to hydrolysis of PIP2 and subsequent IP3-mediated calcium release from the endoplasmic reticulum (ER) (Finch and Augustine, 1998; Takechi et al., 1998; Berridge et al., 2000; Cohen, 2003) (Figure 1). Models 1–7 explored PIP2 signaling and IP3 production (Xu et al., 2003; Hernjak et al., 2005; Brown et al., 2008). Results from Models 4–7 indicated that baseline PIP2 levels are insufficient for requisite IP3 production in the spine, even with apparent anomalous lateral diffusion of PIP2 from the neighboring dendrite (Brown and Loew, 2012). On average, the Purkinje neuron has approximately 14 spines per micron of dendrite (Harris and Stevens, 1988). Each spine is attached to the dendrite branchlet by a neck with diverse morphology (Harris and Stevens, 1989) (see Supplemental Material, S3 IP3R1 in dendritic formation and spine morphology). Model 6 results showed that spine necks of varying radii and lengths also restricted diffusion of produced IP3 out of the spine head (Brown et al., 2008). This supported experimental results from Santamaria et al. with IP3 diffusing more slowly in spiny dendrites than in aspiny dendrites (Santamaria et al., 2006). This suggested that spines might compartmentalize IP3 via spine necks. Simulation results from Model 2 suggested local PIP2 sequestration as a likely source of sufficient PIP2, to fine-tune an experimentally observed (Wang et al., 2000; Sarkisov and Wang, 2008) time window between PF and climbing fiber (CF; from the inferior olive) activation of the Purkinje neuron spine. Stimulation from a single CF innervating the Purkinje neuron cell body and proximal dendrites leads to calcium influx across the plasma membrane, through voltage-gated calcium channels (Ito et al., 1982) (Figure 1). Calcium binding of IP3R1 increases open probability of the receptor (Fiala et al., 1996). IP3R1 serves as the gate-keeper for IP3-induced calcium release. Thus, coincidence detection at IP3R1 leads to more calcium release than with activation of IP3R1 by IP3 or calcium alone (Wang et al., 2000; Hernjak et al., 2005; Ogasawara et al., 2008; Sarkisov and Wang, 2008; Brown et al., 2011).

### “Compensatory pathology” in a mouse model of human SCA1

Mutant Ataxin-1, Ataxin-2, and Ataxin-3 have multiple polyglutamine (CAG, polyQ) repeats (Orr et al., 1993; Kawaguchi et al., 1994; Pulst et al., 1996). Corresponding mouse models exhibit complex behavior with reduced IP3R1 abundance (Lin et al., 2000; Serra et al., 2004; Chou et al., 2008; Hansen et al., 2013) juxtaposed with IP3R1 supersensitivity (Inoue et al., 2001; Serra et al., 2004; Chen et al., 2008; Chou et al., 2008; Brown and Loew, 2012). IP3R1 supersensitivity has been directly shown in SCA2 and SCA3 (Chen et al., 2008; Liu et al., 2009), and speculated in SCA1 (Liu et al., 2009; Kasumu and Bezprozvanny, 2012) (Table 1). All three ataxic mouse models associate with supranormal calcium release (Inoue et al., 2001; Chen et al., 2008; Liu et al., 2009). Models 11 and 12 in the SCA modeling suite support the plausibility of IP3R1 supersensitivity as a necessary component to the Purkinje neuron dysfunction observed in SCA1 (Brown and Loew, 2012).

**Table 1:** **Neurological disorders in humans and mice involving IP3R1, calbindin (CB), parvalbumin (PV), and other calcium signaling proteins**.

**Disorder**	**Mutant protein**	**IP3R1-associated**			**PV/CB**	**References**
		**Expression**	**Sensitivity**	**Direct binding**	**Expression**	
**SMOOTH ENDOPLASMIC RETICULUM CLUSTER**						
SCA1	Ataxin-1	Decreased	M	E	Decreased	Orr et al., 1993; Burright et al., 1995; Lin et al., 2000; Inoue et al., 2001; Liu et al., 2009
SCA2	Ataxin-2	Decreased	Increased	E	Decreased	Pulst et al., 1996; Liu et al., 2009; Hansen et al., 2013
SCA3	Ataxin-3	Decreased	Increased	E	M	Dürr et al., 1996; Paulson et al., 1997; Chen et al., 2008; Chou et al., 2008
SCA7	Ataxin-7	Decreased	M	M	Decreased	David et al., 1997; Friedrich et al., 2012
SCA8	Ataxin-8	M	M	M	M	Moseley et al., 2006; Krysa et al., 2012
SCA17	Ataxin-17	M	M	M	M	Nakamura et al., 2001
HD	Huntingtin	Decreased	Increased	E	M	Datta et al., 2011; Euler et al., 2012
DLPRA	DLPRA	Decreased	M	E	Decreased	Liu et al., 2009; Suzuki et al., 2012
SCA15	IP3R1	Decreased	U	U	U	van de Leemput et al., 2007; Hara et al., 2008; Di Gregorio et al., 2010
SCA16	IP3R1	Decreased	U	U	U	Iwaki et al., 2008
QG	CARP VIII	U^*^	M	E	U^*^	Hirota et al., 2003; Yan et al., 2007; Türkmen et al., 2009
**PLASMA MEMBRANE CLUSTER**						
A-HL	PMCA	Decreased	U	U	U	Kurnellas et al., 2007
ARCCA	mGluR	U	U	U	U	Guergueltcheva et al., 2012
EA2/leaner	Cav2.1	U	U	U	Decreased	Guida et al., 2001; Murchison et al., 2002; Mantuano et al., 2004; Tonelli et al., 2006
SCA6	Cav2.1	U	U	U	U	Ishikawa et al., 1997
SCA14	PKC-γ	U	U	U	U	Alonso et al., 2005; Shuvaev et al., 2011; van Gaalen et al., 2013; Ji et al., 2014
SCA5	Spectrin β	U	U	U	U	Ikeda et al., 2006
EA6	EAAT1	U	U	U	U	de Vries et al., 2009
EA5	CavB4	U	U	U	U	Escayg et al., 2000
EA1	Kv1.1	U	U	U	U	Imbrici et al., 2006
SCA13	Kv3.3	U	U	U	U	Waters et al., 2006
SCA19	Kv4.3	U	U	U	U	Duarri et al., 2012
SCA22	Kv4.3	U	U	U	U	Lee et al., 2012

SCA, spinocerebellar ataxia; HD, Huntington's disease; DLPRA, dentatorubral-pallidoluysian atrophy; leaner, mouse model corresponding to EA2; EA, episodic ataxia; QG, quadrupedal gait ataxia; ARCCA, autosomal-recessive congenital cerebellar ataxia. M, predicted by model; E, experimentally observed; U, Untested. U

* *, tested and unchanged in mouse model very early on at age 14 days, which may be too early to show changes, based on reduced inositol 1,4,5-trisphosphate receptor 1 (IP3R1) expression at age 26 days in the SCA2 mice (Hansen et al., 2013) (Supplemental Material, S4 IP3R1 suppression by CARP). ITPR1, intracellular calcium release channel on the endoplasmic reticulum gated by IP3; PKC-γ, kinase expressed in Purkinje neurons; CARP VIII, Carbonic anhydrase-related protein 8 (CA8), an IP3R1 antagonist (Türkmen et al., 2009); EAAT1, Excitatory amino-acid transporter type 1 (a glutamate transporter); Spectrin β, an excitatory amino-acid transporter type 1 (EAAT4; glutamate transporter) and GluRδ2 (Grid2, glutamate receptor) anchor; DAG, diacylglycerol a product of PLC hydrolysis that activates PKC along with calcium; PIP2, Phosphatidylinositol 4,5-bisphosphate, a plasma membrane phospholipid of the inner leaflet that gives rise to DAG and IP3 when hydrolyzed; PLC, phospholipase C, an enzyme that hydrolyzes PIP2 when activated by G-betagamma from mGluR; mGluR or Grm1, metabotropic glutamate receptor type 1; PMCA, Plasma membrane calcium ATP-ase transports calcium out of the cell; Cav2.1, main P-type calcium channel in PCs, with nonsense/missense mutations causing episodic ataxia type 2 and expansion of CAG repeats causing SCA6; CavB4, accessory subunit that regulates P-type channels encoded by Cav2.1; Kv1.1, Kv3.3, and Kv4.3 are potassium channels that contribute to repolarization of dendritic calcium spikes in Purkinje neurons; A-HL, ataxia and hearing loss in mice, hearing loss in humans. Adapted from Schorge et al. (2010)*.

One of many common denominators for downregulation of calcium homeostasis and glutamatergic signaling proteins in polyQ disorders (e.g., Orr et al., 1993; Kawaguchi et al., 1994; Koide et al., 1994, 1999; Trottier et al., 1994; Pulst et al., 1996; David et al., 1997; Nakamura et al., 2001) is transcription factor retinoid acid receptor-related orphan receptor alpha (RORα) (Serra et al., 2006; Gehrking et al., 2011; Euler et al., 2012). Model 12 results suggest that downregulation of IP3R1, sarcoendoplasmic reticulum calcium ATPase (SERCA), homer, and various other glutamatergic signaling proteins (Serra et al., 2004; Chou et al., 2008) in SCA1, SCA2, and SCA3 may compensate for IP3R1 supersensitivity (Brown and Loew, 2012). IP3R1 expression and sensitivity therefore cannot be considered independently in these polyQ ataxias, but interdependently. Huntington's disease and dentatorubral-pallidoluysian atrophy (DLPRA) are related polyQ disorders with increased IP3R1 sensitivity and reduced IP3R1 abundance (Liu et al., 2009; Datta et al., 2011; Euler et al., 2012; Suzuki et al., 2012). Although the phenotype in these disorders is very different, Model 12 predicts that downregulation of IP3R1 should compensate for supersensitivity. Conversely, Model 12 suggests that downregulation of calcium buffers such as calbindin and parvalbumin worsen pathology with potential feedback and feedforward loops or network motifs (Brown and Loew, 2012) (Supplemental Material, S1 Calcium buffers in SCA and Supplemental Figure S2). This is supported by experimental observations (Vig et al., 1998, 2001). Upregulation of any endogenous inhibitors of IP3R1 should also assist with homeostasis (Supplemental Material, S4 IP3R1 suppression by CARP).

### BK channel in IP3R1-associated ataxia biochemicoelectrophysiological model

IP3R1 interacts closely with the large conductance calcium-activated voltage-gated potassium (BK) channel in glioma cells (Weaver et al., 2007). BK channels appear in lipid rafts in the plasma membrane apposed to the smooth ER (sER). IP3R1 also functionally activates the BK channel in arterial smooth muscle cells (Zhao et al., 2010). It is thought that in other cell types, including neurons, BK channels may form physical complexes with various plasma membrane calcium channels, resulting in a proximity of only a few nanometers from the calcium channel pores (Dai et al., 2009).

In Purkinje neurons, BK channels contribute to repolarization of membrane potential transients in dendrites (Miyasho et al., 2001) and afterhyperpolarization of action potentials at the soma (Sausbier et al., 2004). BK channels are involved in several ataxias that converge on IP3R1-dependent signaling. BK knockout mice are ataxic and show markedly decreased spontaneous firing (tonic and bursting) of Purkinje neurons, with longer interspike intervals due to lack of BK contribution to afterhyperpolarization of the sodium (action potential) spikes which would normally help to reset the sodium channels in wild type mice (Sausbier et al., 2004; Brown and Loew, 2012). BK channels are activated by the P/Q-type calcium channels (Walter et al., 2006), which are mutated in episodic ataxia 2 (EA2) (Guida et al., 2001; Mantuano et al., 2004; Tonelli et al., 2006; Walter et al., 2006) and spinocerebellar ataxia 6 (SCA6) (Ishikawa et al., 1997; Bürk et al., 2014). In the SCA modeling suite (Models 8–9, 13–15), BK channels plays a key role in mediating the effects of IP3R1-mediated calcium release on electrophysiological signals (Brown and Loew, 2012). Combining electrophysiology with detailed biochemistry leads to emergent properties (altered firing of the Purkinje neuron in Models 13–15) that are not possible to simulate in purely electrophysiological or biochemical models (Brown and Loew, 2012).

## Expanding the SCA modeling suite

### Biochemical-electrophysiological modeling

There are other calcium channels that functionally couple with IP3R1. The small conductance calcium-activated potassium (SK) channels are not voltage-gated, but contribute to precision timing (Womack and Khodakhah, 2003; Womack et al., 2004; Walter et al., 2006; Alviña and Khodakhah, 2010a,b). Targeted overactivation of SK channels in SCA2 mice restores regular pacemaking activity (Kasumu et al., 2012). Isolated underactivation of SK channels without a counteracting mutation also yields ataxic mice (Alviña and Khodakhah, 2010a). Addition of this channel to the SCA models (Models 8–9, 13–15) will help mediate the influence of biochemical calcium release on electrophysiology.

### Glutamate receptor modulation by PKC

With insufficient IP3R1, reduced calcium release should result in decreased activation of conventional PKC isoforms. Normally, cytosolic calcium and DAG together activate PKC (Nishizuka, 1984) (Figure 1). PKC phosphorylates α-amino-3-hydroxy-5-methyl-4-isoxazolepropionic acid receptor (AMPAR), another glutamate receptor, and other molecules that are recruited to the plasma membrane and induce AMPAR internalization (Chung et al., 2000, 2003). This depresses Purkinje neuron dendritic spine response to PF stimuli (Figure 1). Human SCA14 involve PKC mutations (Alonso et al., 2005; Shuvaev et al., 2011; van Gaalen et al., 2013; Ji et al., 2014) (Figure 1) (Table 1), which can lead to aberrant AMPAR modulation. Model 16 (“Brown et al., 2012—AMPAR.PKC.PP2A.NO” at www.vcell.org) simulates PKC-AMPAR signaling and can be incorporated into other SCA models (Models 10–15) to investigate SCA14. This PKC-AMPAR sub-model is adapted from simulations by Ogasawara et al. (2007, 2008).

### PKC impact on BK channel activity

Phosphorylation by PKC also inhibits neuronal BK channel activity (Shipston and Armstrong, 1996). Decreased PKC levels could therefore attenuate BK inhibition. This would balance suppression of BK channel activation by lower calcium transients in IP3R1-deficient Purkinje spines. Merging Models 13 and14 with Model 16 could illustrate contributions of BK regulation by PKC phosphorylation to SCAs. Protein kinase A (PKA) (Hall and Armstrong, 2000; Widmer et al., 2003) and PIP2 activation of BK and other potassium channels (Hilgemann et al., 2001; Falkenburger et al., 2010; Zhang et al., 2010) could also be added to these models.

## Potential confirmatory studies to test SCA modeling suite predictions

### SCA mouse models

In addition to SCA1, SCA2, and SCA3 mice (Colomer Gould, 2012; Hansen et al., 2013; Hearst et al., 2014; Switonski et al., 2014), there are other mouse models available for testing SCA modeling suite predictions. The IP3R1^+/−^ mice can most be likened to IP3R1 haploinsufficiency in humans with SCA15/SCA16 (Ogura et al., 2001; van de Leemput et al., 2007; Hara et al., 2008; Iwaki et al., 2008; Di Gregorio et al., 2010; Novak et al., 2010b; Castrioto et al., 2011; Marelli et al., 2011; Obayashi et al., 2012) (Model 10). The ITPR1^opt/opt^ mice also have reduced IP3R1. However, IP3R1 is likely misregulated in these mice, as evidenced by IP3R1-mediated calcium transients that paradoxically show less attenuation to repeated stimulation than wild type mice (Street et al., 1997). If IP3R1 sensitivity is increased in ITPR1^opt/opt^ mice, then these mice could serve as an additional candidate model for polyQ ataxias or other ataxias with decreased expression of supersensitive IP3R1 (see Model 11). Similarly, the reported SCA15 mouse model ITPR1^Δ18/Δ18^ shows reduced levels of IP3R1, but the 18 bp mutation is in the regulatory region of IP3R1 (van de Leemput et al., 2007). Calcium release and membrane electrophysiology need to be probed in these mice to ascertain whether they match the anticipated physiology of SCA15. Human SCA29 has missense mutations in the regulatory domain of IP3R1 (Huang et al., 2012) and would also need such studies in any corresponding mouse model.

### Experimentally available ICpeptides

A number of synthetic experimental peptides resembling sections of the C-terminal of IP3R1 are available for competitive binding in SCA mouse models. The IC4 peptide (also reported as IC1, Q2714-A2749; Tang et al., 2003; Tu et al., 2004) is available for competitive inhibition of PP1α in ataxias with reduced levels of IP3R1 (simulated in Model 10). IP3R1 dephosphorylation by protein phosphatase alpha (PP1α) decreases IP3R1 sensitivity to IP3 (Tang et al., 2003). IC4 (ICpeptide, Figure 1) resembles the tip of the C-terminal of IP3R1 that encodes the PP1α-binding domain. All these peptides can be used to validate and confirm predictions from the SCA modeling suite. The IC-G2736X and IC-10 peptides (Tang et al., 2009) are available for competitive inhibition of mutant Ataxin in ataxias with supersensitive IP3R1 (IC-G2736X simulated in Model 11). Peptide-based therapeutic approaches (Lucchese and Kanduc, 2014) could use viral vectors, as explored for Huntington's disease (HD) (Tang et al., 2009).

### Clinical translation

The SCA modeling suite is poised for continued use in translational studies (Brown et al., 2015). Cerebellar IP3R1 levels (Ogura et al., 2001; van de Leemput et al., 2007) in various SCA mouse models could be experimentally correlated with levels of peripheral lymphocyte IP3R1 from the same mice. The two sets of values could be plotted against each other. Levels of peripheral lymphocyte IP3R1 from ataxic individuals (van de Leemput et al., 2007) could then potentially be compared with corresponding levels in mice to estimate cerebellar levels in humans. In one study of SCA15, Western blot showed variably reduced IP3R1 levels in peripheral lymphocytes from three affected members of the same family relative to an unaffected family member (van de Leemput et al., 2007). Measuring peripheral blood lymphocyte levels of IP3R1 would be relatively noninvasive for humans. Correlated estimates of cerebellar IP3R1 would be useful to help guide therapy, particularly in presymptomatic patients who have undergone genetic testing and counseling (Supplemental Material, S1.6 Presymptomatic staging to consider calbindin modulation).

## Functional consideration of IP3R1 in ataxia

IP3R-mediated calcium release occurs in various tissues (see deSouza et al., 2007; Mondin et al., 2009; Cárdenas et al., 2010; Chen et al., 2010; Ehrlich et al., 2010; Healy et al., 2010; Mandal et al., 2010; Park et al., 2010) including peripheral lymphocytes, but IP3R1 mutations in mice and humans (Matsumoto et al., 1996; Street et al., 1997; Ogura et al., 2001; van de Leemput et al., 2007) (van de Leemput et al., 2007; Hara et al., 2008; Iwaki et al., 2008; Di Gregorio et al., 2010; Huang et al., 2012) lead to primarily cerebellar defects. Nevertheless, the IP3R1^−/−^ (Matsumoto et al., 1996) and IP3R1^opt/opt^ (Street et al., 1997) mice also present with epileptic symptoms, and the conditional cerebellum/brainstem IP3R1 knockout mice and waddles mice present with dystonia (Jiao et al., 2005; Hisatsune et al., 2013) (Supplemental Material, S5 IP3R1 in basal ganglia-independent dystonia).

Most tissues have similar concentrations of all three IP3R isoforms or favor high levels of IP3R2 or IP3R3 over IP3R1 (De Smedt et al., 1997; Tu et al., 2005). Yet, 90% of IP3R in the cerebellar Purkinje neuron is IP3R1 (De Smedt et al., 1997). Insufficient levels of IP3R1 remarkably disrupt Purkinje neuron function, as observed in ataxia (SCA15 van de Leemput et al., 2007; Hara et al., 2008; Iwaki et al., 2008; Di Gregorio et al., 2010; Novak et al., 2010a,b; Marelli et al., 2011).

Other tissues such as smooth muscle, which has 75% of IP3R as IP3R1 (De Smedt et al., 1997), and peripheral lymphocytes, in which the major IP3R isoform is also type 1 (deSouza et al., 2007), likely have compensatory mechanisms involving 25% of IP3R as IP3R2 and IP3R3 to overcome IP3R1 deficits. In addition, there are two regulatory domain phosphorylation site splice variants of IP3R1 (Tu et al., 2002; Wagner et al., 2003). S(II) is favored in the brain (Wagner et al., 2003). Other tissues, such as smooth muscle and peripheral lymphocytes, may differentially phosphorylate their IP3R1 at S(I) (Tang et al., 2003) in response to insufficient levels of the receptor.

Further, there is a high density of sER containing IP3R1 in Purkinje spines (calculated average of ~15% of spine volume from Harris and Stevens, 1988), relative to hippocampal spines (reported as <5% of spine volume from Harris and Stevens, 1989) (Supplemental Material, S6 IP3R1 in hippocampal spines), which are important for synaptic plasticity involved in cognitive learning and memory. This suggests that IP3R1 on sER preferentially serves particular functions in Purkinje spines that may manifest differently in other cell types.

All of these reasons underlie the observation that in IP3R1 mutations, and in several human ataxias with biochemical and electrophysiological signals that converge on IP3R1-dependent signaling (Mikoshiba, 2007; Schorge et al., 2010), the primary clinical manifestation is spinocerebellar ataxia.

## SCA in computational systems neurobiology

Spatial quantitative models have given some insight into how cerebellar Purkinje neuron intracellular processes work together as an efficient system. A computational foundation for studying a wide array of spinocerebellar ataxias that involve mutations in various calcium and potassium channels, kinases, and other molecules, including IP3R1 was developed (Supplemental Material, Supplemental Figure S1). The result is a practical application of Computational Systems Neurobiology (Brown et al., 2012). Using these models to study various ataxias will help us to explain a wide array of experimental observations, elucidate cellular causes of these ataxias in mice and humans, and further understand the relationship between cytosolic calcium and membrane electrophysiology. The SCA modeling suite can help characterize the cellular pathophysiology of IP3R1-associated ataxia. That will help us to understand the biochemical and electrophysiological coupling in excitable membranes, since IP3R1 is highly expressed in the brain, and enriched in the cerebellum (Furuichi et al., 1989; De Smedt et al., 1997; Mikoshiba, 2007).

## Author contributions

Sherry-Ann Brown conceived of, analyzed, designed, drafted, critically revised, approved, and agreed to be accountable for this submitted work. Leslie M. Loew analyzed, designed, drafted, critically revised, approved, and agreed to be accountable for this submitted work.

### Conflict of interest statement

The authors declare that the research was conducted in the absence of any commercial or financial relationships that could be construed as a potential conflict of interest.
