# Supplementary material for: Integration of modeling with experimental and clinical findings synthesizes and refines the central role of inositol 1,4,5-trisphosphate receptor 1 in spinocerebellar ataxia
Source: Front Neurosci. 2015 Jan 21;8:453. doi: 10.3389/fnins.2014.00453 (PMC4300941; doi:10.3389/fnins.2014.00453)
Supplement: Supplementary file 1 [file DataSheet1.DOCX]

**Supplementary Material**

**Inositol 1,4,5-trisphosphate receptor 1 is essential to spinocerebellar ataxia modeling**

**Sherry-Ann Brown^1*^, Leslie M. Loew^2^**

^1^ Department of Medicine, Mayo Clinic, Rochester, MN, USA

^2^ Richard D. Berlin Center for Cell Analysis & Modeling, University of Connecticut Health Center, Farmington, CT, USA

**^*^ Correspondence:** Dr. Sherry-Ann Brown, Mayo Clinic, 200 First Street SW, Rochester, MN, 55905, USA.

brown.sherryann@mayo.edu

1. **Supplementary Data**

**S1 Calcium buffers differentially maintain homeostasis in spinocerebellar ataxia (SCA)**

**S1.1 Calcium buffer downregulation in SCA1 worsens phenotype**

Calcium buffers, such as parvalbumin and calbindin, modify calcium transients under normal conditions (Schwaller, Meyer et al. 2002). In spinocerebellar ataxia 1 (SCA1), expression of these proteins is decreased (Vig, Subramony et al. 1998, Vig, Subramony et al. 2001). This was observed experimentally in SCA1 mouse models and clinically in Purkinje cells from SCA1 patients. Results from SCA computational models suggest that downregulation of these calcium buffers contributes to pathology (Brown and Loew 2012) (Fig. S2a). This has been supported by a number of experiments. First, crossing SCA1 mice with calbindin knockout mice further worsens the ataxic phenotype (Vig, Subramony et al. 2001). Second, peak calcium amplitudes and average spine lengths are two times higher in parvalbumin/calbindin combined knockout mouse model Purkinje neurons compared with wild type mice (Vecellio, Schwaller et al. 2000, Schmidt, Stiefel et al. 2003).

**S1.2 Supranormal calcium from calbindin downregulation obligates supersensitive IP3R1**

In SCA1, inositol trisphosphate receptor type 1 (IP3R1) is downregulated before calbindin (Lin, Antalffy et al. 2000). SCA computational model results suggest that decreased concentrations of IP3R1 in SCA1, SCA2, and SCA3 may partially compensate for supersensitivity (Brown and Loew 2012) (Fig. S2b). Schorge et al suggest that prior to onset of symptoms, calcium buffering might obscure the deficit in IP3R1 signaling (Schorge, van de Leemput et al. 2010). Calbindin quells supranormal calcium transients, but eventually this buffering capability is lost (Fig. S2b). Yet, SCA computational models indicate that downregulation of buffering capability is not sufficient to cause symptoms, only if supersensitive IP3R1 is assumed (Brown and Loew 2012). IP3R1 sensitivity has been shown experimentally in SCA2 (Liu, Tang et al. 2009) and SCA3 (Chen, Tang et al. 2008) and implied in SCA1 (Liu, Tang et al. 2009). It is not clear why calbindin is not downregulated sooner in SCA1 mice. Calbindin activity is also suppressed by aggregation and cross-linkage with mutant Ataxin-1 in the nucleus (Vig, Wei et al. 2007). This is mediated by tissue transglutaminase type 2, likely even before observable downregulation. It is not clear why calbindin is not downregulated sooner in SCA1 mice. There may be many common denominators for downregulation of calcium homeostasis and signaling proteins in polyQ disorders (e.g., (Orr, Chung et al. 1993, Kawaguchi, Okamoto et al. 1994, Koide, Ikeuchi et al. 1994, Trottier, Biancalana et al. 1994, Pulst, Nechiporuk et al. 1996, David, Abbas et al. 1997, Koide, Kobayashi et al. 1999, Nakamura, Jeong et al. 2001)). One common denominator is the transcription factor retinoid acid receptor-related orphan receptor alpha (RORα), present in the nucleus (Serra, Duvick et al. 2006, Gehrking, Andresen et al. 2011, Euler, Friedrich et al. 2012).

**S1.3 Calbindin is neuroprotective in SCA1**

Recently, Vig et al assessed changes in calbindin localization and parvalbumin expression in response to proposed potential therapy in SCA1 mice (Vig, Hearst et al. 2014). Specifically, Acid-Sensing Ion Channel 1a (ASIC1a) knockout mice were developed to suppress disease in SCA1 mice. ASIC1a knockout (KO) SCA1 mice exhibited increased translocation of cerebellar Purkinje neuron calbindin from the nucleus to the cystosol. This was different from the usual combined cystolic and nuclear calbindin localization in SCA1 mice with wild type ASIC1a. Upregulation of parvalbumin was also noted in KO SCA1 mice. Increased availability of cytosolic calbindin and parvalhumin was associated with improved morphology of cerebellar Purkinje neurons, in comparison to to age-matched SCA1 mice with wild type ASIC1a. KO SCA1 mice also displayed improved motor coordination and reduction in cerebellar Purkinje neuron loss. Thus, ASIC1a was implicated as a mediator of SCA1 pathophysiology and calcium buffer dysregulation.

In another recent study, Hearst et al assessed calbindin expression in response to another potential therapy for SCA1 (Hearst, Shao et al. 2014). Focused laser light induced hyperthermia (HT) was used to induce expression of molecular chaperone heat-shock proteins (HSPs). HSPs are known to modulate polyglutamine protein aggregation and afford neuroprotection (Cummings, Sun et al. 2001). Mild in vivo application of HT in SCA1 mice increased temperature and induced significant cerebellar Hsp70 production. There was no evidence of neuronal damage or inflammatory response. Repeated exposure led to higher levels of calbindin. Subsequent improvement in cerebellar Purkinje neuron morphology and suppression of disease was observed.

Taken together, these two new studies indicate that higher levels of calcium buffers are neuroprotective. This is consistent with findings of symptom onset following calcium buffer downregulation in SCA1 as well as SCA2 and SCA3 mice (Schorge, van de Leemput et al. 2010)(Chou, Yeh et al. 2008, Hansen, Meera et al. 2013). This is also consistent with SCA modeling results.

**S1.4 Calbindin downregulation in a mouse model of human SCA2**

Calbindin is downregulated in SCA2 mice at 4-6 weeks of age, before IP3R1 and other proteins prior to symptom onset (Kinoshita-Kawada, Oberdick et al. 2004, Kato, Knierman et al. 2012, Hansen, Meera et al. 2013) (Fig. S2c). This may be due to a number of observations. Originally thought of as purely a calcium buffer, calbindin also participates in buffered transport and calcium sensing influenced by synaptic activation (Schmidt 2012). The majority of calcium that diffuses from the activated spine to the adjacent dendrite is bound to calbindin (Schmidt, Kunerth et al. 2007, Schmidt and Eilers 2009). In addition, calbindin interacts directly with various proteins whose functions depend on spine calcium concentration (Schmidt 2012). Calcium buffering and transport by calbindin are already included in the SCA modeling suite (Models 1-7, 9-16). The 3D geometries in the SCA modeling suite (Models 4-6, 15) can be used to simulate spatiotemporal mobility of calbindin from the post-synaptic density to downstream effector proteins, and the effect this has on calcium signaling and membrane electrophysiology in SCA mice.

**S1.5 Late compensation by other proteins in SCA2**

Glutamate receptor δ2 (Grid2), metabotropic glutamate receptor 1 (mGluR), and Purkinje cell protein 2 (Pcp2) are downregulated at 8 weeks of age, prior to IP3R1 downregulation at 24 weeks (Hansen, Meera et al. 2013). Decreased levels of Grid2 lead to increased surface expression of available mGluR (Kato, Knierman et al. 2012). Low levels of PCP2 inhibit the P-type calcium channels, the predominant class of plasma membrane calcium channels in the Purkinje neuron spiny dendrites (Kinoshita-Kawada, Oberdick et al. 2004). Thus, in spite of (or more likely in response to) early calbindin downregulation at 6 weeks, several calcium signaling and glutamatergic molecules are downregulated at 8 weeks to modulate intracellular calcium concentration. Subsequent IP3R1 downregulation at 24 weeks in SCA2 mice (Hansen, Meera et al. 2013) (as opposed to 5 weeks in SCA1 mice (Serra, Byam et al. 2004)) may be a late strategy for compensation (Fig. S2c). Nevertheless, SCA2 mice are still ataxic as the balance between compensation (explored in Model 12) and pathology rests in favor of pathology.

**S1.6 Presymptomatic staging to consider calbindin modulation**

Presymptomatic mouse data is particularly useful in the context of clinical studies of presymptomatic patients. A recent paper by Schuler-Faccini et al explored presymptomatic testing (PST) and genetic counseling in Brazil and Portugal (Schuler-Faccini, Osorio et al. 2014). Patients at risk of developing SCA3 received guidance in learning more about the disease and the decision-making process. Additionally, Velázquez-Pérez et al recently delineated the prodromal stage of SCA2 (Velázquez-Pérez, Rodríguez-Labrada et al. 2014, Velázquez-Pérez, Rodríguez-Labrada et al. 2014). Early features were closely examined in presymptomatic patients. These studies help develop our knowledge of the various stages of early SCA disease progression. This will assist in determining timing of therapeutics to modulate calbindin and potentially delay symptom onset.

**S1.7 Differential effect of calbindin on homeostasis timing in SCAs**

Certain caveats must be considered, primarily the differential effect of calcium buffers on homeostasis timing in SCAs. Differential regulation of calbindin in SCA1 and SCA2 was noted above. Further, calbindin downregulation in SCA1 likely has the opposite effect of downregulation in the leaner mouse model (Murchison, Dove et al. 2002) of human Episodic Ataxia type 2 (EA2). In EA2, calbindin downregulation would be expected to deter symptom onset by augmenting free calcium to compensate for pathologically reduced density and function of mutated P-type calcium channels (Murchison, Dove et al. 2002). Calbindin in ataxias, as well as general pathophysiology of EA2 and spinocerebellar ataxia 6 (SCA6) (Ishikawa, Tanaka et al. 1997, Bürk, Kaiser et al. 2014), could further be assessed in the SCA 3D computational models (Brown and Loew 2012).

**S1.8 Calbindin in Network Motifs**

Figure S2d illustrates proposed interconnected network motifs present in calcium and glutamatergic signaling. These include feedback loops that involve calbindin. Figure S2e shows generalized universal biological network motifs in systems biology (Alon 2007). The presence of such feedback suggests limitations of the traditional reductionist view in biology to understand these complex interactions in the Purkinje neuron. Translational systems biology should therefore be used to model and increase our understanding of the function of network motifs in ataxia. This interplay among humans, mice, computer models, and simulations contributes to what Thongboonkerd describes as the promise and challenge of systems biology in translational medicine (Thongboonkerd 2013).

**S2 Calcium-induced calcium release crosstalk**

Liu et al (Liu, Tang et al. 2009) and Chen et (Chen, Tang et al. 2008) found that long-term feeding of SCA2 and SCA3 mice with dantrolene improved motor coordination (see Fig. 1). Dantrolene inhibits ryanodine receptor (RYR), a calcium channel present on sER (Fruen, Mickelson et al. 1997, Zhao, Li et al. 2001, Gerbershagen, Fiege et al. 2003, Krause, Gerbershagen et al. 2004) (see Fig. 1). IP3R1 functionally couples with ryanodine receptor type 1 (RYR1) in adult mammalian skeletal muscle to form calcium sparks (Tjondrokoesoemo, Li et al. 2013). Crosstalk between IP3R and RYR is also implicated in cardiomyocytes and smooth muscle cells (Gordienko and Bolton 2002, Zhang, Yip et al. 2003). RYR1 is present in Purkinje dendrites, but not in spines (Sharp, McPherson et al. 1993). IP3R1-mediated calcium from the spine cytosol diffuses through the spine neck, leading to activation of adjacent dendritic RYR1 (Khodakhah and Armstrong 1997)(see Fig. 1). Thus, dantrolene administration to SCA2 and SCA3 mice likely attenuates calcium in dendrites adjacent to activated spines. This improves summated function of the Purkinje neuron. Addition of RYR1 to the dendrite in Model 11 would facilitate study of alternative therapeutics for SCAs.

Intracellular calcium also induces store-operated calcium entry (SOCE) through plasma membrane channels (Deak, Blass et al. 2014). IP3R1-mediated calcium release from the sER activates stromal interacting molecule 1 (STIM 1), which oligomerizes and migrates to ER-plasma membrane junctions. STIM1 binds to Orai by a STIM-Orai activating region (SOAR), leading to activation and opening of Orai, the plasma membrane calcium channel portion of SOCE (Deak, Blass et al. 2014, Hendron, Wang et al. 2014, Wang, Wang et al. 2014). Stim and Orai together are thought to form store-operated channels (SOCs) that mediate this capacitative calcium entry (CCE) (Michaelis, Nieswandt et al. 2014). These SOCs functionally (and to some degree physically) couple with IP3R1 at the sER-plasma membrane junction (Mikoshiba and Hattori 2000).

Thus, IP3R1-mediated calcium release leads to calcium-induced calcium release crosstalk through channels on both the sER and the plasma membrane.

**S3 IP3R1 in dendritic formation and spine morphology**

IP3R1 is important for Purkinje dendrite formation and morphology, with mice totally deficient of IP3R1 showing Purkinje neuron degeneration and poorly formed spiny dendrites, with absence of spines (Mikoshiba, Huchet et al. 1979, Furuichi, Yoshikawa et al. 1989, Maeda, Niinobe et al. 1989). IP3R1 plays a crucial role specifically in parallel fiber-Purkinje spine synapses, with abnormal rearrangement of these synaptic circuits and paradoxically hyperspiny dendrites in Purkinje neuron-specific IP3R1 knockout mice, resulting in loss of LTD and severe ataxia (Sugawara, Hisatsune et al. 2013). In addition, IP3R1 in cerebellar granule cells helps regulate the morphology of Purkinje dendrites and parallel fiber-Purkinje spine synapses, by increasing BDNP expression in response to activation of α-amino-3-hydroxy-5-methyl-4-isoxazolepropionic acid receptor (AMPAR) and mGluR (Hisatsune, Kuroda et al. 2006).

Other proteins in the Purkinje neuron spine modulate IP3R1 localization and stabilization in the spine, possibly contributing to regulation of spine morphology. Homer and Myosin Va are thought to help guide and maintain IP3R1-containing sER in the Purkinje spine, and their differential expression in SCA1 mice relative to wild type may be partially compensatory for supersensitive IP3R1 (Lin, Antalffy et al. 2000, Miyata, Finch et al. 2000, Wagner and Hammer 2003, Serra, Byam et al. 2004, Brown and Loew 2012). The protein 4.1N, which contains binding regions for actin, Spectrin, AMPAR, **N-methyl-D-aspartate receptor** (NMDAR), and IP3R1, facilitates the functional coupling of IP3R1 with cytosolic actin to restrict lateral diffusion of IP3R1 in the sER membrane and has no effect on IP3R3 (Fukatsu, Bannai et al. 2004, Fukatsu, Bannai et al. 2006, Fukatsu, Bannai et al. 2010). These findings support the centrol role of IP3R1 in interacting signaling pathways important for Purkinje dendrite spine dynamics and morphology.

**S4 IP3R1 suppression by CARP**

In normal Purkinje neuron spines, endogenous inhibitors limit IP3R1 affinity for inositol trisphosphate (IP3) relative to other cell types (Watras, Orlando et al. 2000, Hirota, Ando et al. 2003, Watras, Fink et al. 2005, Ando, Mizutani et al. 2006). The carbonic anhydrase-related protein (CARP) is a more recently discovered endogenous IP3R1 inhibitor that is also mutated in human ataxia and corresponding mouse models (Kato 1990, Kelly, Nógrádi et al. 1994, Nógrádi, Jonsson et al. 1997, Hirota, Ando et al. 2003, Jiao, Yan et al. 2005, Yan, Jiao et al. 2007, Türkmen, Guo et al. 2009, Kaya, Aldhalaan et al. 2011) (see subtype CARP VIII listed in Table 1), and is targeted as an autoantigen in melanoma-associated paraneoplastic cerebellar degeneration (Bataller, Sabater et al. 2004). CARP is a non-catalytic relative of the zinc metalloenzyme carbonic anhydrases, and is composed of carbonic anhydrase-related protein VIII (CARP VIII; CA8; Car8), carbonic anhydrase-related protein X (CARP X; Car10), and carbonic anhydrase-related protein XI (CARP XI; Car11) in vertebrates (Aspatwar, Tolvanen et al. 2013, Aspatwar, Tolvanen et al. 2014). CARP VIII is predominantly expressed in cerebellar Purkinje neurons, alongside lower levels of CARP X and XI (Taniuchi, Nishimori et al. 2002, Taniuchi, Nishimori et al. 2002, Nishimori, Takeuchi et al. 2003, Aspatwar, Tolvanen et al. 2010). Subcellular localization coincides with IP3R1, suggesting that CARP VIII is part of an IP3R1 signaling complex, along with Homer and other calcium signaling related proteins (Mikoshiba 2007).

The N-terminal of CARP VIII includes a short stretch of glutamic acid trinucleotide repeats (16 GAG repeats), which is much shorter than in other SCA polyQ proteins, such as Ataxin-1, Ataxin-2, and Ataxin-3 that have CAG repeats (Kelly, Nógrádi et al. 1994). This polyQ region is not found in other carbonic anhydrases or CARPs, and is included in the minimum binding site for IP3R1 (amino acids 45-291 of CARP VIII) (Kelly, Nógrádi et al. 1994, Hirota, Ando et al. 2003, Aspatwar, Tolvanen et al. 2010). This suggests that cerebellar ataxias with unknown genetic causes could be probed to look for any evidence of GAG repeat expansion in CARP as a potential underlying cause, similar to polyQ SCAs, due to its effect on IP3R1-mediated calcium release (Aspatwar, Tolvanen et al. 2010).

The spontaneous mouse models ‘waddles’ and ‘lurcher’ and human QG ataxia do not have repeat expansions, but instead reduced expression of CARP VIII (Kato 1990, Kelly, Nógrádi et al. 1994, Nógrádi, Jonsson et al. 1997, Hirota, Ando et al. 2003, Jiao, Yan et al. 2005, Yan, Jiao et al. 2007, Türkmen, Guo et al. 2009, Kaya, Aldhalaan et al. 2011). This should lead to supersensitive IP3R1, as in the polyQ ataxias (Chen, Tang et al. 2008, Liu, Tang et al. 2009), but has not yet been tested. One would expect the converse to be true – high levels of CARP VIII leading to low affinity of IP3R1 to IP3. However, in SCA3, overexpression of CARP VIII juxtaposed with mutant Ataxin-3 (which induces supersensitive IP3R1) leads to a delicate balance that results in IP3R1 supersensitivity (Hsieh, Chang et al. 2013). Perhaps in the future the expression of CARP VIII can be therapeutically fine-tuned in waddles, lurcher, and SCA3 mice, then ultimately in QG ataxia and SCA3 in humans to help normalize calcium release and membrane electrophysiology, with improved phenotype.

In SCA1-SCA3, differential expression of glutamatergic signaling proteins is thought to have a partially compensatory effect (Brown and Loew 2012). However, IP3R1, calbindin, parvalbumin, and glutamatergic signaling genes are not shown to be differentially expressed early on at two weeks in the waddles mouse model, which has a 19-bp loss-of-function deletion mutation in exon 8 of CARP VIII (Jiao, Yan et al. 2005, Yan, Jiao et al. 2007). Instead, downregulation of cell division, zinc-ion binding, synapse integrity, and synaptic plasticity genes, and upregulation of Golgi apparatus genes and other signaling and downstream calcium-sensing genes such as Hippocalcin-like protein 1 and solute carrier family 8 (sodium/calcium exchanger) member 1, are noted. This does not rule out the possibility of IP3R1 downrelagulation for partial compensation. IP3R1 is downregulated much later on at 26 weeks in SCA2 mice (Hansen, Meera et al. 2013), which suggests that IP3R1 expression should be further examined beyond two weeks in cerebella from CARP VIII-deficient mice.

Interestingly, CARP forms part of a multifunctional signaling macrocomplex hinged on IP3R1, with other IP3R1 binding partners including homer (class of scaffolding proteins), protein 4.1N, huntingtin-associated protein-1A, protein phosphatases (PPI and PP2A), receptor for activated C kinase 1 (RACK1), ankyrin, chromogranin, inositol trisphosphate receptor-binding protein released with inositol trisphosphate (IRBIT), Na,K-ATPase, and endoplasmic reticulum resident protein 44 (ERp44) (Mikoshiba 2007). The function of the macrocomplex depends on the unique properties of each binding protein. For example, at physiological IP3 concentrations, the binding of IP3 to IP3R1 releases IRBIT from IP3R1 with downstream signaling activity, while IRBIT is thought to inhibit by IP3R1 by competitively binding the IP3-binding site (Ando, Mizutani et al. 2003, Ando, Mizutani et al. 2006, Ando, Kawaai et al. 2014).

CARP also contributes to dendritic morphology, based on several observations in CARP VIII-deficient mice (Nógrádi, Jonsson et al. 1997, Hirasawa, Xu et al. 2007). In these mice, climbing fiber terminals extend into distal dendrites, which have sparse parallel fiber terminals. Many of the distal dendritic spines are well formed, but without synaptic contact. The spines with synaptic contact exhibit limited spontaneous excitatory neurotransmission in response to parallel fiber activation. This is in contrast to wild type mice, in which climbing fiber terminals are restricted to the soma and proximal dendrites, while distal dendrite synapses are preferentially activated by parallel fibers. In the mutant mice therefore, CARP VIII deficiency leads to structural and functional aberrancies involving synaptic morphology and excitation. This demonstrates the role of CARP VIII in synaptogenesis, maintenance of distal dendritic morphology, parallel fiber synapse stabilization, restriction of climbing fiber innervation to proximal dendrites, and parallel fiber activation of Purkinje neurons. This is mediated by intracellular calcium, as relatively high calcium concentrations in Purkinje neuron spines, for example, from climbing fiber activation inhibits expansion of parallel fiber territory (Sotelo, Hillman et al. 1975, Bravin, Morando et al. 1999). CARP VIII also likely helps to fine-tune parallel fiber innervation in wild type mice, since inhibition of calcium transients results in extension of parallel fiber terminals to proximal dendrites that are usually innervated by climbing fibers alone (Sotelo, Hillman et al. 1975, Bravin, Morando et al. 1999).

Given all of these contributions to cerebellar Purkinje neurons structure and function, CARP could be studied in the SCA modeling suite, by adjusting IP3R1 affinity (d_IP3_) to IP3.

**S5 IP3R1 in basal ganglia-independent dystonia**

Dystonia involves sustained contractions of agonistic and antagonistic muscles simultaneously, inducing twisting and repetitive movements and abnormal posturing (Hisatsune, Miyamoto et al. 2013). Traditionally thought to be a basal ganglia disorder, there is now evidence that dystonia can occur independent of the basal ganglia, with implication of cerebellar Purkinje neurons (Zhuang, Li et al. 2004, Yan, Jiao et al. 2007, Hisatsune, Miyamoto et al. 2013). A mouse model with conditional IP3R1 knockout in the cerebellum and brainstem revealed specific coupling between Purkinje neuron firing and dystonia (Hisatsune, Miyamoto et al. 2013). These dystonic movements were not dependent on the basal ganglia, and resolved with inhibition of Purkinje neurons. A different mouse model (waddles), also exhibits appendicular dystonia, supporting the role of CARP in the Purkinje neuron as a modulator of motor control (Yan, Jiao et al. 2007).

**S6 IP3R1 in hippocampal spines**

IP3R1 is involved in synaptic plasticity in the cerebellum, and also in the hippocampus. In the hippocampus, several authors have shown that IP3R1 is integral to effecting long-term depression and modulating long-term potentiation important for learning and memory (Fujii, Matsumoto et al. 2000, Itoh, Ito et al. 2001, Taufiq, Fujii et al. 2005, Yamazaki, Sugihara et al. 2011). These examined stimulation of CA1, CA3, and mossy fiber regions of the hippocampus, using short and standard tetanus along with low-frequency stimuli. In one study, standard tetanus (100 pulses at 100 Hz) delivered to wild type mouse hippocampal CA1 neurons induced long-term potentiation (LTP), while low-frequency stimuli (1000 pulses at 1 Hz) induced long-term depression (LTD) (Fujii, Matsumoto et al. 2000). Delivery of low-frequency stimulation one hour after standard tetanus lead to depotentiation, during which LTP was reversed, whereas delivery of low-frequency stimulation one hour before standard tetanus suppressed LTP induction. Using IP3R1-deficient mice, those authors found that LTP suppression and depotentiation were dependent on IP3R1. This is analogous to a time window important for coincidence detection mediated by IP3R1-mediated calcium release in the cerebellar Purkinje neuron (Brown, Morgan et al. 2008, Sarkisov and Wang 2008). In the hippocampus, LTP induction is also dependent on IP3R1 (Fujii, Matsumoto et al. 2000, Nishiyama, Hong et al. 2000, Itoh, Ito et al. 2001, Taufiq, Fujii et al. 2005, Yamazaki, Sugihara et al. 2011). LTD is also dependent on IP3R1, particularly heterosynaptic LTD (Nishiyama, Hong et al. 2000, Itoh, Ito et al. 2001, Taufiq, Fujii et al. 2005). Taken together, these studies suggest that IP3R1 plays an important role in attenuation of synaptic excitation in hippocampal CA1 and CA3 neurons. This manifests as LTP suppression, LTP depotentiation, raising the threshold for induction of LTP, and facilitating LTD.

**Supplementary Figures and Tables**

## Supplementary Tables

**Supplemental Table S1 Public models composing the Spinocerebellar Ataxia Modeling Suite**

| **Model Name** | **No.** | **Software** | **Database** | **Ref.** |
| --- | --- | --- | --- | --- |
| Purkinje_compartmental | 1 | Virtual Cell | Virtual Cell | (Hernjak, Slepchenko et al. 2005) |
| Purkinje Compartmental – adapted from Hernjak et al | 2 | Virtual Cell | Virtual Cell | (Brown, Morgan et al. 2008) |
| Purkinje 1D | 3 | Virtual Cell | Virtual Cell | (Brown, Morgan et al. 2008) |
| Purkinje 3D – 12 PF Stimuli | 4 | Virtual Cell | Virtual Cell | (Brown, Morgan et al. 2008) |
| Purkinje 3D – 4 PF Stimuli | 5 | Virtual Cell | Virtual Cell | (Brown, Morgan et al. 2008) |
| Purkinje 3D Several Spines –12 PF Stimuli | 6 | Virtual Cell | Virtual Cell | (Brown, Morgan et al. 2008) |
| PIP2 Diffusion 1D and 3D | 7 | Virtual Cell | Virtual Cell | (Brown, Morgan et al. 2008) |
| A simplified cerebellar Purkinje neuron (the PPR model) | 8 | NEURON | ModelDb | (Brown, Moraru et al. 2011) |
| Brown et al. 2010 Purkinje MultiCompartmental Combined Biochem & Electrophysiol | 9 | Virtual Cell | Virtual Cell | (Brown, Moraru et al. 2011) |
| Brown et al. 2011 Analysis of SCA15-SCA16 with IC4 Peptide Application | 10 | Virtual Cell | Virtual Cell | (Brown and Loew 2012) |
| Brown et al. 2011 Analysis of SCA1-SCA2-SCA3 with IC-G2736X Peptide | 11 | Virtual Cell | Virtual Cell | (Brown and Loew 2012) |
| Brown et al. 2011 SCA1 Compensation Analysis | 12 | Virtual Cell | Virtual Cell | (Brown and Loew 2012) |
| Brown et al. 2011 Purkinje Biochem-Electrophysiol SCA | 13 | Virtual Cell | Virtual Cell | (Brown and Loew 2012) |
| Brown et al. 2011 Combined Purkinje - Several Spines - Current Injection | 14 | Virtual Cell | Virtual Cell | (Brown and Loew 2012) |
| Brown et al. 2011 Purkinje 3D Spine – Spinocerebellar Ataxia study | 15 | Virtual Cell | Virtual Cell | (Brown and Loew 2012) |
| Brown et al. 2012 - AMPAR.PKC.PP2A.NO | 16 | Virtual Cell | Virtual Cell | -- |

## Supplementary Figures

**Supplemental Figure S1 The SCA modeling suite has 16 Virtual NEURON models.** The suite of models was created as a unifying framework to determine the interdependence of various calcium signaling and ion channel molecules in SCAs (Brown and Loew 2012)(Fig. 1). The component models (Models 1-16) are listed in Table S1. Simulations can be accessed as “Shared BioModels” and a “Shared MathModel” under the usernames Brown and Hernjak, by logging in to the Virtual Cell (VCell) modeling and simulation software available at http://www.vcell.org. Model 1 simulates pulsatile increases in inositol trisphosphate (IP3) concentration leading to a rise in intracellular calcium (Hernjak, Slepchenko et al. 2005). Model 2 features the physiological production of IP3 from hydrolysis of phosphatidylinositol-4,5-bisphosphate (PIP2) and link the appropriate biochemical IP3 signal to IP3R1-mediated calcium release in cerebellar Purkinje spines (Brown, Morgan et al. 2008). Activation by 4 or 12 PFs followed by a single climbing fiber (CF) stimulus was modeled in order to simulate published Purkinje spine stimulation patterns (Finch and Augustine 1998, Wang, Denk et al. 2000, Hernjak, Slepchenko et al. 2005, Brown, Morgan et al. 2008). Model 3 represents the same physiology in one dimension, with the spine cytosol described by a discrete length along the one dimensional geometry representing the rest of the spiny dendrite (Brown, Morgan et al. 2008). Models 4 and 5 are three dimensional renditions in an experimentally derived geometry (Brown, Morgan et al. 2008). Model 6 has stimulus from 12 parallel fibers that activate 6 different Purkinje spines (Brown, Morgan et al. 2008). Model 7 has the same one and three dimensional features as Models 3 and 4, and focuses on restricted diffusion of membrane-bound PIP2 through spine necks of varying radii and lengths (Brown, Morgan et al. 2008). Model 8, the preserved path reduction (PPR) model, reduces the entire Purkinje neuron to equivalent cylinders with preservation electrophysiological activity, with a dedicated path from the soma to the spine {Brown, 2010, Virtual NEURON: a strategy for merged biochemical and electrophysiological modeling}(Brown, Moraru et al. 2011). The PPR model allows for reproduction in Virtual Cell for combination with biochemical signaling, leading to Model 9, a biochemicoelectrophysiological model (Brown, Moraru et al. 2011). Models 8 and 9 are Virtual NEURONs, computationally economical models for merged biochemical and electrophysiological analysis of the cerebellar Purkinje neuron and are publicly available in NEURON on ModelDb (http://senselab.med.yale.edu/modeldb/) and in Virtual Cell (www.vcell.org), respectively. Models 10-15 form the core of modeling SCA pathophysiology (Brown and Loew 2012). Model 10 reflects the cell biology of SCA15 and SCA16, along with application of a peptide that resembles the C-terminal of IP3R1 and could potentially serve a therapeutic role. Model 11 reflects the pathophysiology of SCA1, SCA2, and SCA3, with application of a different therapeutic peptide suitable for these disorders. Model 12 analyzes the compensatory effects of downregulation of various calcium signaling molecules in SCA1 in the setting of supranormal intracellular calcium release. Models 13 and 14 explore potential pathophysiological effects of SCA on coupled biochemical and electrophysiological modeling. Model 15 provides a 3D spine for spatial SCA modeling. Model 16 replicates well-mixed biochemical interactions among α-amino-3-hydroxy-5-methylisoxazole-4-propionic acid subtype glutamate receptors (AMPAR), protein kinase C (PKC), protein phosphatase type 2 alpha (PP2α), and nitric oxide (NO) in the Purkinje spiny dendrite, based on estimations by Ogasawara et al (Ogasawara, Doi et al. 2007, Ogasawara, Doi et al. 2008). Integration of Model 16 with other SCA suite models will allow for further investigation of the relationships among membrane-bound receptors and ion channels and cytosolic molecules.

**Supplemental Figure S2 Impact of calcium buffers in SCA1 and SCA2**. (A), Downregulation of metabotropic glutamate receptor 1 (mGluR1), inositol trisphosphate receptor type 1 (IP3R1), sarcoplasmic/endoplasmic reticulum CA2+-ATPase type 3 (SERCA3), and Homer increases compensation for IP3R1 supersensitivity; calbindin downregulation worsens SCA1 phenotype (Brown and Loew 2012), which pivots about the homeostasis fulcrum with loss of compensation; all this precedes symptom onset. (B), Calbindin downregulation also precedes symptom onset in SCA2. Downregulation of Purkinje cell protein 2 (PCP2), mGluR1 (Grm1), Glutamate receptor δ2 (Grid2), and IP3R1 much later contribute to compensation but do not shift the balance from pathology towards compensation. (C), Downregulation of calcium buffers (e.g., calbindin) worsens calcium signal dysregulation, and contributes to symptom onset. (D), At a glance, calcium signaling in Purkinje neuron spines and dendrites may incorporate various network motifs, including coherent and incoherent FFLs, feedback loops, and DORs. (E), One family of network motifs is the feed forward loop (FFL), which appears in hundreds of gene systems in several organisms; three key interactors are involved in this motif: a regulator, X, which regulates Y, and Z, which is regulated by both X and Y; in the coherent FFL, both arms act in a concerted fashion, while in the incoherent FFL, the two arms act in opposition; another motif is the dense overlapping regulon (DOR), which involves many inputs regulating many outputs; adapted from (Alon 2007). Ca, calcium; Calbindin, calcium-binding protein; mluR or Grm1, metabotropic glutamate receptor on the plasma membrane of Purkinje neuron spines and dendrites; IMPase, myo-inositol monophosphatase, an enzyme that dephosphorylates and thereby inactivates members of the IP3 degradation pathway; DAG, diacylglycerol a product of PLC hydrolysis that activates PKC along with calcium; PKC, protein kinase C expressed in Purkinje neurons that helps control expression of surface molecules; RYR, ryanodine receptor, a transporter from calcium exit from the ER to the cytosol; Cav2.1, the main P-type calcium channel in Purkinje neurons; IP3R, inositol trisphosphate (IP3) receptor, intracellular calcium release channel on the endoplasmic reticulum gated by IP3. R1, (Schmidt 2012); R2, (McCudden, Hains et al. 2005).

**3 References**

Alon, U. (2007). "Network motifs: theory and experimental approaches." Nat Rev Genet **8**(6): 450-461.

Ando, H., K. Kawaai and K. Mikoshiba (2014). "IRBIT: a regulator of ion channels and ion transporters." Biochim Biophys Acta **1843**(10): 2195-2204.

Ando, H., A. Mizutani, H. Kiefer, D. Tsuzurugi, T. Michikawa and K. Mikoshiba (2006). "IRBIT suppresses IP3 receptor activity by competing with IP3 for the common binding site on the IP3 receptor." Mol Cell **22**(6): 795-806.

Ando, H., A. Mizutani, T. Matsu-ura and K. Mikoshiba (2003). "IRBIT, a novel inositol 1,4,5-trisphosphate (IP3) receptor-binding protein, is released from the IP3 receptor upon IP3 binding to the receptor." J Biol Chem **278**(12): 10602-10612.

Aspatwar, A., M. E. Tolvanen, C. Ortutay and S. Parkkila (2010). "Carbonic anhydrase related protein VIII and its role in neurodegeneration and cancer." Curr Pharm Des **16**(29): 3264-3276.

Aspatwar, A., M. E. Tolvanen, C. Ortutay and S. Parkkila (2014). "Carbonic anhydrase related proteins: molecular biology and evolution." Subcell Biochem **75**: 135-156.

Aspatwar, A., M. E. Tolvanen and S. Parkkila (2010). "Phylogeny and expression of carbonic anhydrase-related proteins." BMC Mol Biol **11**: 25.

Aspatwar, A., M. E. Tolvanen and S. Parkkila (2013). "An update on carbonic anhydrase-related proteins VIII, X and XI." J Enzyme Inhib Med Chem **28**(6): 1129-1142.

Bataller, L., L. Sabater, A. Saiz, C. Serra, B. Claramonte and F. Graus (2004). "Carbonic anhydrase-related protein VIII: autoantigen in paraneoplastic cerebellar degeneration." Ann Neurol **56**(4): 575-579.

Bravin, M., L. Morando, A. Vercelli, F. Rossi and P. Strata (1999). "Control of spine formation by electrical activity in the adult rat cerebellum." Proc Natl Acad Sci U S A **96**(4): 1704-1709.

Brown, S., F. Morgan, J. Watras and L. Loew (2008). "Analysis of phosphatidylinositol-4,5-bisphosphate signaling in cerebellar Purkinje spines." Biophys J **95**(4): 1795-1812.

Brown, S.-A., F. Morgan, J. Watras and L. M. Loew (2008). Analysis of Phosphatidylinositol-4,5-bisphosphate Signaling in Cerebellar Purkinje Spines.

Brown, S. A. and L. M. Loew (2012). "Computational analysis of calcium signaling and membrane electrophysiology in cerebellar Purkinje neurons associated with ataxia." BMC Syst Biol **6**(1): 70.

Brown, S. A., I. I. Moraru, J. C. Schaff and L. M. Loew (2011). "Virtual NEURON: a strategy for merged biochemical and electrophysiological modeling." J Comput Neurosci.

Bürk, K., F. J. Kaiser, S. Tennstedt, L. Schöls, F. R. Kreuz, T. Wieland, T. M. Strom, T. Büttner, R. Hollstein, D. Braunholz, J. Plaschke, G. Gillessen-Kaesbach and C. Zühlke (2014). "A novel missense mutation in CACNA1A evaluated by in silico protein modeling is associated with non-episodic spinocerebellar ataxia with slow progression." Eur J Med Genet **57**(5): 207-211.

Chen, X., T. Tang, H. Tu, O. Nelson, M. Pook, R. Hammer, N. Nukina and I. Bezprozvanny (2008). "Deranged calcium signaling and neurodegeneration in spinocerebellar ataxia type 3." J Neurosci **28**(48): 12713-12724.

Chou, A., T. Yeh, P. Ouyang, Y. Chen, S. Chen and H. Wang (2008). "Polyglutamine-expanded ataxin-3 causes cerebellar dysfunction of SCA3 transgenic mice by inducing transcriptional dysregulation." Neurobiol Dis **31**(1): 89-101.

Cummings, C., Y. Sun, P. Opal, B. Antalffy, R. Mestril, H. Orr, W. Dillmann and H. Zoghbi (2001). "Over-expression of inducible HSP70 chaperone suppresses neuropathology and improves motor function in SCA1 mice." Hum Mol Genet **10**(14): 1511-1518.

David, G., N. Abbas, G. Stevanin, A. Dürr, G. Yvert, G. Cancel, C. Weber, G. Imbert, F. Saudou, E. Antoniou, H. Drabkin, R. Gemmill, P. Giunti, A. Benomar, N. Wood, M. Ruberg, Y. Agid, J. Mandel and A. Brice (1997). "Cloning of the SCA7 gene reveals a highly unstable CAG repeat expansion." Nat Genet **17**(1): 65-70.

Deak, A. T., S. Blass, M. J. Khan, L. N. Groschner, M. Waldeck-Weiermair, S. Hallström, W. F. Graier and R. Malli (2014). "IP3-mediated STIM1 oligomerization requires intact mitochondrial Ca2+ uptake." J Cell Sci **127**(Pt 13): 2944-2955.

Euler, P., B. Friedrich, R. Ziegler, A. Kuhn, K. S. Lindenberg, C. Weiller and B. Zucker (2012). "Gene expression analysis on a single cell level in Purkinje cells of Huntington's disease transgenic mice." Neurosci Lett **517**(1): 7-12.

Finch, E. and G. Augustine (1998). "Local calcium signalling by inositol-1,4,5-trisphosphate in Purkinje cell dendrites." Nature **396**(6713): 753-756.

Fruen, B., J. Mickelson and C. Louis (1997). "Dantrolene inhibition of sarcoplasmic reticulum Ca2+ release by direct and specific action at skeletal muscle ryanodine receptors." J Biol Chem **272**(43): 26965-26971.

Fujii, S., M. Matsumoto, K. Igarashi, H. Kato and K. Mikoshiba (2000). "Synaptic plasticity in hippocampal CA1 neurons of mice lacking type 1 inositol-1,4,5-trisphosphate receptors." Learn Mem **7**(5): 312-320.

Fukatsu, K., H. Bannai, T. Inoue and K. Mikoshiba (2006). "4.1N binding regions of inositol 1,4,5-trisphosphate receptor type 1." Biochem Biophys Res Commun **342**(2): 573-576.

Fukatsu, K., H. Bannai, T. Inoue and K. Mikoshiba (2010). "Lateral diffusion of inositol 1,4,5-trisphosphate receptor type 1 in Purkinje cells is regulated by calcium and actin filaments." J Neurochem **114**(6): 1720-1733.

Fukatsu, K., H. Bannai, S. Zhang, H. Nakamura, T. Inoue and K. Mikoshiba (2004). "Lateral diffusion of inositol 1,4,5-trisphosphate receptor type 1 is regulated by actin filaments and 4.1N in neuronal dendrites." J Biol Chem **279**(47): 48976-48982.

Furuichi, T., S. Yoshikawa, A. Miyawaki, K. Wada, N. Maeda and K. Mikoshiba (1989). "Primary structure and functional expression of the inositol 1,4,5-trisphosphate-binding protein P400." Nature **342**(6245): 32-38.

Gehrking, K. M., J. M. Andresen, L. Duvick, J. Lough, H. Y. Zoghbi and H. T. Orr (2011). "Partial loss of Tip60 slows mid-stage neurodegeneration in a spinocerebellar ataxia type 1 (SCA1) mouse model." Hum Mol Genet **20**(11): 2204-2212.

Gerbershagen, M., M. Fiege, T. Krause, K. Agarwal and F. Wappler (2003). "[Dantrolene. Pharmacological and therapeutic aspects]." Anaesthesist **52**(3): 238-245.

Gordienko, D. V. and T. B. Bolton (2002). "Crosstalk between ryanodine receptors and IP(3) receptors as a factor shaping spontaneous Ca(2+)-release events in rabbit portal vein myocytes." J Physiol **542**(Pt 3): 743-762.

Hansen, S. T., P. Meera, T. S. Otis and S. M. Pulst (2013). "Changes in Purkinje cell firing and gene expression precede behavioral pathology in a mouse model of SCA2." Hum Mol Genet **22**(2): 271-283.

Hearst, S. M., Q. Shao, M. Lopez, D. Raucher and P. J. Vig (2014). "Focused Cerebellar Laser Light Induced Hyperthermia Improves Symptoms and Pathology of Polyglutamine Disease SCA1 in a Mouse Model." Cerebellum.

Hendron, E., X. Wang, Y. Zhou, X. Cai, J. I. Goto, K. Mikoshiba, Y. Baba, T. Kurosaki, Y. Wang and D. L. Gill (2014). "Potent functional uncoupling between STIM1 and Orai1 by dimeric 2-aminodiphenyl borinate analogs." Cell Calcium **56**(6): 482-492.

Hernjak, N., B. Slepchenko, K. Fernald, C. Fink, D. Fortin, I. Moraru, J. Watras and L. Loew (2005). "Modeling and analysis of calcium signaling events leading to long-term depression in cerebellar Purkinje cells." Biophys J **89**(6): 3790-3806.

Hirasawa, M., X. Xu, R. B. Trask, T. P. Maddatu, B. A. Johnson, J. K. Naggert, P. M. Nishina and A. Ikeda (2007). "Carbonic anhydrase related protein 8 mutation results in aberrant synaptic morphology and excitatory synaptic function in the cerebellum." Mol Cell Neurosci **35**(1): 161-170.

Hirota, J., H. Ando, K. Hamada and K. Mikoshiba (2003). "Carbonic anhydrase-related protein is a novel binding protein for inositol 1,4,5-trisphosphate receptor type 1." The Biochemical journal **372**(Pt 2): 435-441.

Hisatsune, C., Y. Kuroda, T. Akagi, T. Torashima, H. Hirai, T. Hashikawa, T. Inoue and K. Mikoshiba (2006). "Inositol 1,4,5-trisphosphate receptor type 1 in granule cells, not in Purkinje cells, regulates the dendritic morphology of Purkinje cells through brain-derived neurotrophic factor production." J Neurosci **26**(42): 10916-10924.

Hisatsune, C., H. Miyamoto, M. Hirono, N. Yamaguchi, T. Sugawara, N. Ogawa, E. Ebisui, T. Ohshima, M. Yamada, T. K. Hensch, M. Hattori and K. Mikoshiba (2013). "IP3R1 deficiency in the cerebellum/brainstem causes basal ganglia-independent dystonia by triggering tonic Purkinje cell firings in mice." Front Neural Circuits **7**: 156.

Hsieh, M., W. H. Chang, C. F. Hsu, I. Nishimori, C. L. Kuo and T. Minakuchi (2013). "Altered expression of carbonic anhydrase-related protein XI in neuronal cells expressing mutant ataxin-3." Cerebellum **12**(3): 338-349.

Ishikawa, K., H. Tanaka, M. Saito, N. Ohkoshi, T. Fujita, K. Yoshizawa, T. Ikeuchi, M. Watanabe, A. Hayashi, Y. Takiyama, M. Nishizawa, I. Nakano, K. Matsubayashi, M. Miwa, S. Shoji, I. Kanazawa, S. Tsuji and H. Mizusawa (1997). "Japanese families with autosomal dominant pure cerebellar ataxia map to chromosome 19p13.1-p13.2 and are strongly associated with mild CAG expansions in the spinocerebellar ataxia type 6 gene in chromosome 19p13.1." Am J Hum Genet **61**(2): 336-346.

Itoh, S., K. Ito, S. Fujii, K. Kaneko, K. Kato, K. Mikoshiba and H. Kato (2001). "Neuronal plasticity in hippocampal mossy fiber-CA3 synapses of mice lacking the inositol-1,4,5-trisphosphate type 1 receptor." Brain Res **901**(1-2): 237-246.

Jiao, Y., J. Yan, Y. Zhao, L. R. Donahue, W. G. Beamer, X. Li, B. A. Roe, M. S. Ledoux and W. Gu (2005). "Carbonic anhydrase-related protein VIII deficiency is associated with a distinctive lifelong gait disorder in waddles mice." Genetics **171**(3): 1239-1246.

Kato, A. S., M. D. Knierman, E. R. Siuda, J. T. Isaac, E. S. Nisenbaum and D. S. Bredt (2012). "Glutamate receptor δ2 associates with metabotropic glutamate receptor 1 (mGluR1), protein kinase Cγ, and canonical transient receptor potential 3 and regulates mGluR1-mediated synaptic transmission in cerebellar Purkinje neurons." J Neurosci **32**(44): 15296-15308.

Kato, K. (1990). "Sequence of a novel carbonic anhydrase-related polypeptide and its exclusive presence in Purkinje cells." FEBS Lett **271**(1-2): 137-140.

Kawaguchi, Y., T. Okamoto, M. Taniwaki, M. Aizawa, M. Inoue, S. Katayama, H. Kawakami, S. Nakamura, M. Nishimura and I. Akiguchi (1994). "CAG expansions in a novel gene for Machado-Joseph disease at chromosome 14q32.1." Nat Genet **8**(3): 221-228.

Kaya, N., H. Aldhalaan, B. Al-Younes, D. Colak, T. Shuaib, F. Al-Mohaileb, A. Al-Sugair, M. Nester, S. Al-Yamani, A. Al-Bakheet, N. Al-Hashmi, M. Al-Sayed, B. Meyer, H. Jungbluth and M. Al-Owain (2011). "Phenotypical spectrum of cerebellar ataxia associated with a novel mutation in the CA8 gene, encoding carbonic anhydrase (CA) VIII." Am J Med Genet B Neuropsychiatr Genet **156B**(7): 826-834.

Kelly, C., A. Nógrádi, R. Walker, K. Caddy, J. Peters and N. Carter (1994). "Lurching, reeling, waddling and staggering in mice--is carbonic anhydrase (CA) VIII a candidate gene?" Biochem Soc Trans **22**(3): 359S.

Khodakhah, K. and C. M. Armstrong (1997). "Inositol trisphosphate and ryanodine receptors share a common functional Ca2+ pool in cerebellar Purkinje neurons." Biophys J **73**(6): 3349-3357.

Kinoshita-Kawada, M., J. Oberdick and M. Xi Zhu (2004). "A Purkinje cell specific GoLoco domain protein, L7/Pcp-2, modulates receptor-mediated inhibition of Cav2.1 Ca2+ channels in a dose-dependent manner." Brain Res Mol Brain Res **132**(1): 73-86.

Koide, R., T. Ikeuchi, O. Onodera, H. Tanaka, S. Igarashi, K. Endo, H. Takahashi, R. Kondo, A. Ishikawa and T. Hayashi (1994). "Unstable expansion of CAG repeat in hereditary dentatorubral-pallidoluysian atrophy (DRPLA)." Nat Genet **6**(1): 9-13.

Koide, R., S. Kobayashi, T. Shimohata, T. Ikeuchi, M. Maruyama, M. Saito, M. Yamada, H. Takahashi and S. Tsuji (1999). "A neurological disease caused by an expanded CAG trinucleotide repeat in the TATA-binding protein gene: a new polyglutamine disease?" Hum Mol Genet **8**(11): 2047-2053.

Krause, T., M. Gerbershagen, M. Fiege, R. Weisshorn and F. Wappler (2004). "Dantrolene--a review of its pharmacology, therapeutic use and new developments." Anaesthesia **59**(4): 364-373.

Lin, X., B. Antalffy, D. Kang, H. Orr and H. Zoghbi (2000). "Polyglutamine expansion down-regulates specific neuronal genes before pathologic changes in SCA1." Nat Neurosci **3**(2): 157-163.

Liu, J., T. Tang, H. Tu, O. Nelson, E. Herndon, D. Huynh, S. Pulst and I. Bezprozvanny (2009). "Deranged calcium signaling and neurodegeneration in spinocerebellar ataxia type 2." J Neurosci **29**(29): 9148-9162.

Maeda, N., M. Niinobe, Y. Inoue and K. Mikoshiba (1989). "Developmental expression and intracellular location of P400 protein characteristic of Purkinje cells in the mouse cerebellum." Dev Biol **133**(1): 67-76.

McCudden, C. R., M. D. Hains, R. J. Kimple, D. P. Siderovski and F. S. Willard (2005). "G-protein signaling: back to the future." Cell Mol Life Sci **62**(5): 551-577.

Michaelis, M., B. Nieswandt, D. Stegner, J. Eilers and R. Kraft (2014). "STIM1, STIM2, and Orai1 regulate store-operated calcium entry and purinergic activation of microglia." Glia.

Mikoshiba, K. (2007). "IP3 receptor/Ca2+ channel: from discovery to new signaling concepts." Journal of neurochemistry **102**(5): 1426-1446.

Mikoshiba, K. and M. Hattori (2000). "IP3 receptor-operated calcium entry." Sci STKE **2000**(51): pe1.

Mikoshiba, K., M. Huchet and J. P. Changeux (1979). "Biochemical and immunological studies on the P400 protein, a protein characteristic of the Purkinje cell from mouse and rat cerebellum." Dev Neurosci **2**(6): 254-275.

Miyata, M., E. Finch, L. Khiroug, K. Hashimoto, S. Hayasaka, S. Oda, M. Inouye, Y. Takagishi, G. Augustine and M. Kano (2000). "Local calcium release in dendritic spines required for long-term synaptic depression." Neuron **28**(1): 233-244.

Murchison, D., L. Dove, L. Abbott and W. Griffith (2002). "Homeostatic compensation maintains Ca2+ signaling functions in Purkinje neurons in the leaner mutant mouse." Cerebellum **1**(2): 119-127.

Nakamura, K., S. Jeong, T. Uchihara, M. Anno, K. Nagashima, T. Nagashima, S. Ikeda, S. Tsuji and I. Kanazawa (2001). "SCA17, a novel autosomal dominant cerebellar ataxia caused by an expanded polyglutamine in TATA-binding protein." Hum Mol Genet **10**(14): 1441-1448.

Nishimori, I., H. Takeuchi, Morimoto, K. Taniuchi, N. Okamoto, S. Onishi and Y. Ohtsuki (2003). "Expression of carbonic anhydrase-related protein VIII, X and XI in the enteric autonomic nervous system." Biomed Res **14**(1): 69-73.

Nishiyama, M., K. Hong, K. Mikoshiba, M. M. Poo and K. Kato (2000). "Calcium stores regulate the polarity and input specificity of synaptic modification." Nature **408**(6812): 584-588.

Nógrádi, A., N. Jonsson, R. Walker, K. Caddy, N. Carter and C. Kelly (1997). "Carbonic anhydrase II and carbonic anhydrase-related protein in the cerebellar cortex of normal and lurcher mice." Brain Res Dev Brain Res **98**(1): 91-101.

Ogasawara, H., T. Doi, K. Doya and M. Kawato (2007). "Nitric oxide regulates input specificity of long-term depression and context dependence of cerebellar learning." PLoS Comput Biol **3**(1): e179.

Ogasawara, H., T. Doi and M. Kawato (2008). "Systems biology perspectives on cerebellar long-term depression." Neurosignals **16**(4): 300-317.

Orr, H., M. Chung, S. Banfi, T. J. Kwiatkowski, A. Servadio, A. Beaudet, A. McCall, L. Duvick, L. Ranum and H. Zoghbi (1993). "Expansion of an unstable trinucleotide CAG repeat in spinocerebellar ataxia type 1." Nat Genet **4**(3): 221-226.

Pulst, S., A. Nechiporuk, T. Nechiporuk, S. Gispert, X. Chen, I. Lopes-Cendes, S. Pearlman, S. Starkman, G. Orozco-Diaz, A. Lunkes, P. DeJong, G. Rouleau, G. Auburger, J. Korenberg, C. Figueroa and S. Sahba (1996). "Moderate expansion of a normally biallelic trinucleotide repeat in spinocerebellar ataxia type 2." Nat Genet **14**(3): 269-276.

Sarkisov, D. and S. Wang (2008). "Order-dependent coincidence detection in cerebellar Purkinje neurons at the inositol trisphosphate receptor." J Neurosci **28**(1): 133-142.

Schmidt, H. (2012). "Three functional facets of calbindin D-28k." Front Mol Neurosci **5**: 25.

Schmidt, H. and J. Eilers (2009). "Spine neck geometry determines spino-dendritic cross-talk in the presence of mobile endogenous calcium binding proteins." J Comput Neurosci **27**(2): 229-243.

Schmidt, H., S. Kunerth, C. Wilms, R. Strotmann and J. Eilers (2007). "Spino-dendritic cross-talk in rodent Purkinje neurons mediated by endogenous Ca2+-binding proteins." J Physiol **581**(Pt 2): 619-629.

Schmidt, H., K. M. Stiefel, P. Racay, B. Schwaller and J. Eilers (2003). "Mutational analysis of dendritic Ca2+ kinetics in rodent Purkinje cells: role of parvalbumin and calbindin D28k." J Physiol **551**(Pt 1): 13-32.

Schorge, S., J. van de Leemput, A. Singleton, H. Houlden and J. Hardy (2010). "Human ataxias: a genetic dissection of inositol triphosphate receptor (ITPR1)-dependent signaling." Trends Neurosci.

Schuler-Faccini, L., C. M. Osorio, F. Romariz, M. Paneque, J. Sequeiros and L. B. Jardim (2014). "Genetic counseling and presymptomatic testing programs for Machado-Joseph Disease: lessons from Brazil and Portugal." Genet Mol Biol **37**(1 Suppl): 263-270.

Schwaller, B., M. Meyer and S. Schiffmann (2002). "'New' functions for 'old' proteins: the role of the calcium-binding proteins calbindin D-28k, calretinin and parvalbumin, in cerebellar physiology. Studies with knockout mice." Cerebellum **1**(4): 241-258.

Serra, H., C. Byam, J. Lande, S. Tousey, H. Zoghbi and H. Orr (2004). "Gene profiling links SCA1 pathophysiology to glutamate signaling in Purkinje cells of transgenic mice." Hum Mol Genet **13**(20): 2535-2543.

Serra, H., L. Duvick, T. Zu, K. Carlson, S. Stevens, N. Jorgensen, A. Lysholm, E. Burright, H. Zoghbi, H. Clark, J. Andresen and H. Orr (2006). "RORalpha-mediated Purkinje cell development determines disease severity in adult SCA1 mice." Cell **127**(4): 697-708.

Sharp, A. H., P. S. McPherson, T. M. Dawson, C. Aoki, K. P. Campbell and S. H. Snyder (1993). "Differential immunohistochemical localization of inositol 1,4,5-trisphosphate- and ryanodine-sensitive Ca2+ release channels in rat brain." J Neurosci **13**(7): 3051-3063.

Sotelo, C., D. E. Hillman, A. J. Zamora and R. Llinás (1975). "Climbing fiber deafferentation: its action on Purkinje cell dendritic spines." Brain Res **98**(3): 574-581.

Sugawara, T., C. Hisatsune, T. D. Le, T. Hashikawa, M. Hirono, M. Hattori, S. Nagao and K. Mikoshiba (2013). "Type 1 inositol trisphosphate receptor regulates cerebellar circuits by maintaining the spine morphology of purkinje cells in adult mice." J Neurosci **33**(30): 12186-12196.

Taniuchi, K., I. Nishimori, T. Takeuchi, K. Fujikawa-Adachi, Y. Ohtsuki and S. Onishi (2002). "Developmental expression of carbonic anhydrase-related proteins VIII, X, and XI in the human brain." Neuroscience **112**(1): 93-99.

Taniuchi, K., I. Nishimori, T. Takeuchi, Y. Ohtsuki and S. Onishi (2002). "cDNA cloning and developmental expression of murine carbonic anhydrase-related proteins VIII, X, and XI." Brain Res Mol Brain Res **109**(1-2): 207-215.

Taufiq, A. M., S. Fujii, Y. Yamazaki, H. Sasaki, K. Kaneko, J. Li, H. Kato and K. Mikoshiba (2005). "Involvement of IP3 receptors in LTP and LTD induction in guinea pig hippocampal CA1 neurons." Learn Mem **12**(6): 594-600.

Thongboonkerd, V. (2013). "The promise and challenge of systems biology in translational medicine." Clin Sci (Lond) **124**(6): 389-390.

Tjondrokoesoemo, A., N. Li, P. H. Lin, Z. Pan, C. J. Ferrante, N. Shirokova, M. Brotto, N. Weisleder and J. Ma (2013). "Type 1 inositol (1,4,5)-trisphosphate receptor activates ryanodine receptor 1 to mediate calcium spark signaling in adult Mammalian skeletal muscle." J Biol Chem **288**(4): 2103-2109.

Trottier, Y., V. Biancalana and J. Mandel (1994). "Instability of CAG repeats in Huntington's disease: relation to parental transmission and age of onset." J Med Genet **31**(5): 377-382.

Türkmen, S., G. Guo, M. Garshasbi, K. Hoffmann, A. J. Alshalah, C. Mischung, A. Kuss, N. Humphrey, S. Mundlos and P. N. Robinson (2009). "CA8 mutations cause a novel syndrome characterized by ataxia and mild mental retardation with predisposition to quadrupedal gait." PLoS Genet **5**(5): e1000487.

Vecellio, M., B. Schwaller, M. Meyer, W. Hunziker and M. R. Celio (2000). "Alterations in Purkinje cell spines of calbindin D-28 k and parvalbumin knock-out mice." Eur J Neurosci **12**(3): 945-954.

Velázquez-Pérez, L., R. Rodríguez-Labrada, N. Canales-Ochoa, J. M. Montero, G. Sánchez-Cruz, R. Aguilera-Rodríguez, L. E. Almaguer-Mederos and J. M. Laffita-Mesa (2014). "Progression of early features of spinocerebellar ataxia type 2 in individuals at risk: a longitudinal study." Lancet Neurol **13**(5): 482-489.

Velázquez-Pérez, L., R. Rodríguez-Labrada, E. M. Cruz-Rivas, J. Fernández-Ruiz, I. Vaca-Palomares, J. Lilia-Campins, B. Cisneros, A. Peña-Acosta, Y. Vázquez-Mojena, R. Diaz, J. J. Magaña-Aguirre, T. Cruz-Mariño, A. Estupiñán-Rodríguez, J. M. Laffita-Mesa, R. González-Piña, N. Canales-Ochoa and Y. González-Zaldivar (2014). "Comprehensive Study of Early Features in Spinocerebellar Ataxia 2: Delineating the Prodromal Stage of the Disease." Cerebellum.

Vig, P., S. Subramony, E. Burright, J. Fratkin, D. McDaniel, D. Desaiah and Z. Qin (1998). "Reduced immunoreactivity to calcium-binding proteins in Purkinje cells precedes onset of ataxia in spinocerebellar ataxia-1 transgenic mice." Neurology **50**(1): 106-113.

Vig, P., S. Subramony and D. McDaniel (2001). "Calcium homeostasis and spinocerebellar ataxia-1 (SCA-1)." Brain Res Bull **56**(3-4): 221-225.

Vig, P. J., S. M. Hearst, Q. Shao and M. E. Lopez (2014). "Knockdown of Acid-Sensing Ion Channel 1a (ASIC1a) Suppresses Disease Phenotype in SCA1 Mouse Model." Cerebellum **13**(4): 479-490.

Vig, P. J., J. Wei, Q. Shao, M. D. Hebert, S. H. Subramony and L. T. Sutton (2007). "Role of tissue transglutaminase type 2 in calbindin-D28k interaction with ataxin-1." Neurosci Lett **420**(1): 53-57.

Wagner, W. and J. r. Hammer (2003). "Myosin V and the endoplasmic reticulum: the connection grows." J Cell Biol **163**(6): 1193-1196.

Wang, S., W. Denk and M. Häusser (2000). "Coincidence detection in single dendritic spines mediated by calcium release." Nat Neurosci **3**(12): 1266-1273.

Wang, X., Y. Wang, Y. Zhou, E. Hendron, S. Mancarella, M. D. Andrake, B. S. Rothberg, J. Soboloff and D. L. Gill (2014). "Distinct Orai-coupling domains in STIM1 and STIM2 define the Orai-activating site." Nat Commun **5**: 3183.

Watras, J., C. Fink and L. Loew (2005). "Endogenous inhibitors of InsP3-induced Ca2+ release in neuroblastoma cells." Brain Res **1055**(1-2): 60-72.

Watras, J., R. Orlando and I. Moraru (2000). "An endogenous sulfated inhibitor of neuronal inositol trisphosphate receptors." Biochemistry **39**(12): 3452-3460.

Yamazaki, Y., T. Sugihara, J. Goto, K. Chida, H. Fujiwara, K. Kaneko, S. Fujii and K. Mikoshiba (2011). "Role of inositol 1, 4, 5-trisphosphate receptors in the postsynaptic expression of guinea pig hippocampal mossy fiber depotentiation." Brain Res **1387**: 19-28.

Yan, J., Y. Jiao, F. Jiao, J. Stuart, L. R. Donahue, W. G. Beamer, X. Li, B. A. Roe, M. S. LeDoux and W. Gu (2007). "Effects of carbonic anhydrase VIII deficiency on cerebellar gene expression profiles in the wdl mouse." Neurosci Lett **413**(3): 196-201.

Zhang, W. M., K. P. Yip, M. J. Lin, L. A. Shimoda, W. H. Li and J. S. Sham (2003). "ET-1 activates Ca2+ sparks in PASMC: local Ca2+ signaling between inositol trisphosphate and ryanodine receptors." Am J Physiol Lung Cell Mol Physiol **285**(3): L680-690.

Zhao, F., P. Li, S. Chen, C. Louis and B. Fruen (2001). "Dantrolene inhibition of ryanodine receptor Ca2+ release channels. Molecular mechanism and isoform selectivity." J Biol Chem **276**(17): 13810-13816.

Zhuang, P., Y. Li and M. Hallett (2004). "Neuronal activity in the basal ganglia and thalamus in patients with dystonia." Clin Neurophysiol **115**(11): 2542-2557.
