# Supplementary figures and images for: Integration of modeling with experimental and clinical findings synthesizes and refines the central role of inositol 1,4,5-trisphosphate receptor 1 in spinocerebellar ataxia
Source: Front Neurosci. 2015 Jan 21;8:453. doi: 10.3389/fnins.2014.00453 (PMC4300941; doi:10.3389/fnins.2014.00453)

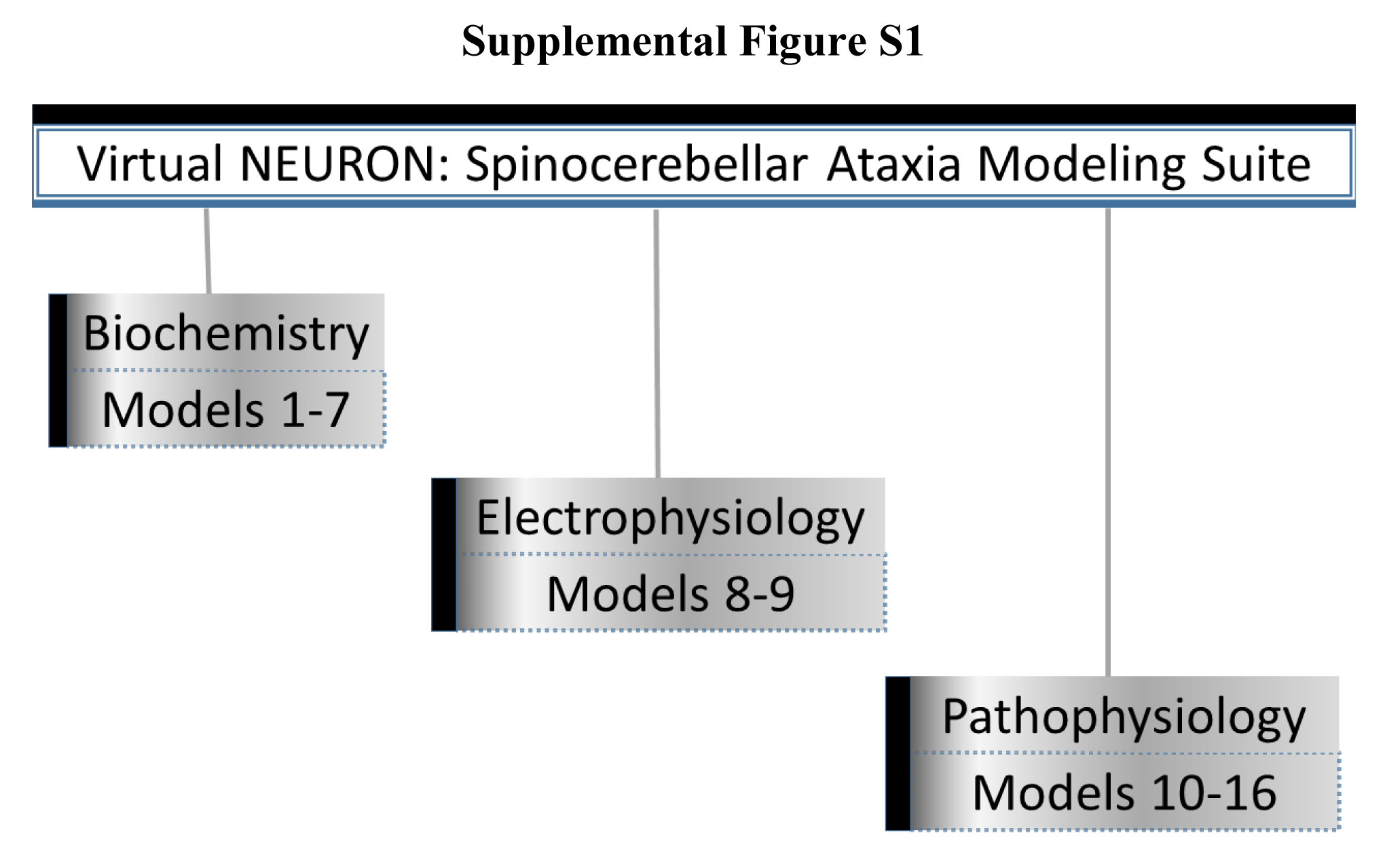

Supplement: Supplementary file 2 [file Image1.TIF]

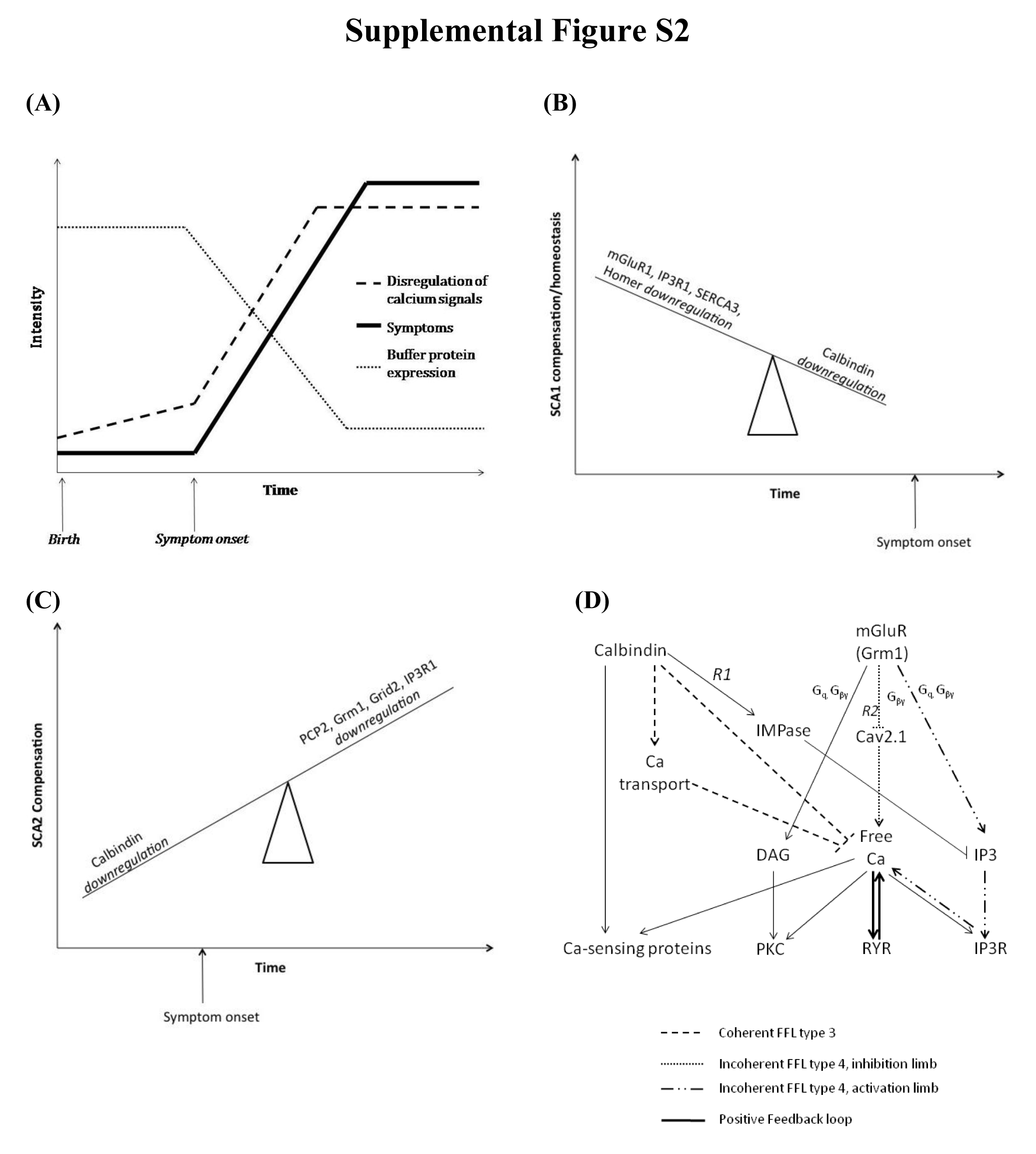

Supplement: Supplementary file 3 [file Image2.TIF]

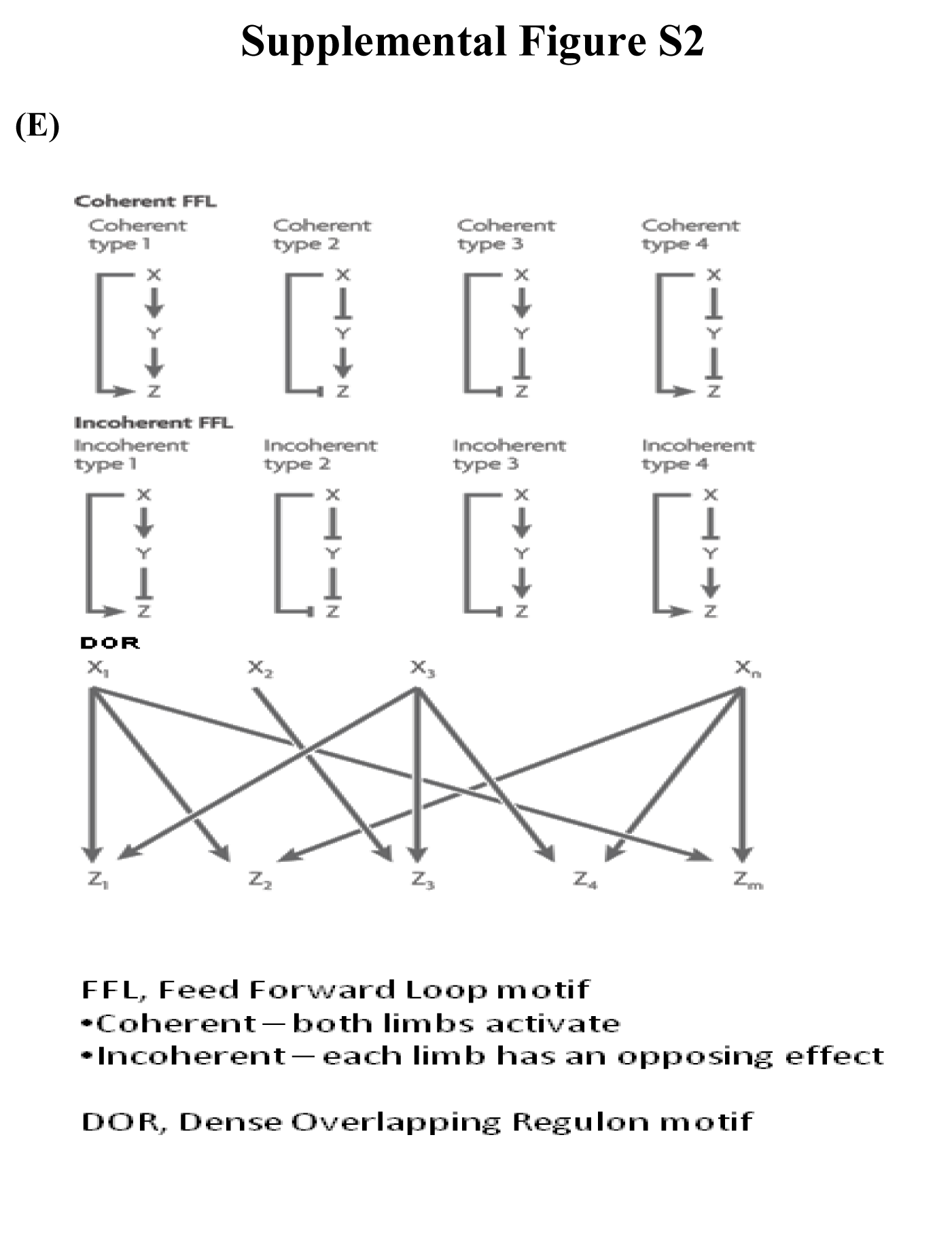

Supplement: Supplementary file 4 [file Image3.TIF]
